# Transcriptional signature of early cisplatin drug-tolerant persister cells in lung adenocarcinoma

**DOI:** 10.3389/fonc.2023.1208403

**Published:** 2023-10-17

**Authors:** Rodolfo Chavez-Dominguez, Dolores Aguilar-Cazares, Mario Perez-Medina, Santiago Avila-Rios, Maribel Soto-Nava, Alfonso Mendez-Tenorio, Lorenzo Islas-Vazquez, Jesus J. Benito-Lopez, Miriam Galicia-Velasco, Jose S. Lopez-Gonzalez

**Affiliations:** ^1^ Departamento de Enfermedades Cronico-Degenerativas, Laboratorio de Cancer Pulmonar, Instituto Nacional de Enfermedades Respiratorias, Ismael Cosio Villegas, Ciudad de Mexico, Mexico; ^2^ Posgrado en Ciencias Biologicas, Universidad Nacional Autonoma de Mexico, Ciudad de Mexico, Mexico; ^3^ Escuela Nacional de Ciencias Biologicas, Instituto Politecnico Nacional, Ciudad de Mexico, Mexico; ^4^ Centro de Investigacion en Enfermedades Infecciosas, Instituto Nacional de Enfermedades Respiratorias Ismael Cosio Villegas, Ciudad de Mexico, Mexico; ^5^ Laboratorio de Biotecnologia y Bioinformatica Genomica, Departamento de Bioquimica, Escuela Nacional de Ciencias Biologicas, Instituto Politecnico Nacional, Ciudad de Mexico, Mexico; ^6^ Departamento de Inmunologia y Unidad de Investigacion, Instituto de Oftalmologia “Conde de Valenciana”, Ciudad de Mexico, Mexico

**Keywords:** lung cancer, non-small cell lung carcinoma, lung adenocarcinoma, cisplatin, chemotherapy resistance, intrinsic resistance, drug-tolerant persister cells, SOCS1

## Abstract

Resistance to cisplatin is the main cause of treatment failure in lung adenocarcinoma. Drug-tolerant-persister (DTP) cells are responsible for intrinsic resistance, since they survive the initial cycles of treatment, representing a reservoir for the emergence of clones that display acquired resistance. Although the molecular mechanisms of DTP cells have been described, few studies have investigated the earliest molecular alterations of DTP cells in intrinsic resistance to cisplatin. In this work, we report a gene expression signature associated with the emergence of cisplatin-DTP cells in lung adenocarcinoma cell lines. After a single exposure to cisplatin, we sequenced the transcriptome of cisplatin-DTPs to identify differentially expressed genes. Bioinformatic analysis revealed that early cisplatin-DTP cells deregulate metabolic and proliferative pathways to survive the drug insult. Interaction network analysis identified three highly connected submodules in which SOCS1 had a significant participation in controlling the proliferation of cisplatin-DTP cells. Expression of the candidate genes and their corresponding protein was validated in lung adenocarcinoma cell lines. Importantly, the expression level of SOCS1 was different between CDDP-susceptible and CDDP-resistant lung adenocarcinoma cell lines. Moreover, knockdown of SOCS1 in the CDDP-resistant cell line partially promoted its susceptibility to CDDP. Finally, the clinical relevance of the candidate genes was analyzed *in silico*, according to the overall survival of cisplatin-treated patients from The Cancer Genome Atlas. Survival analysis showed that downregulation or upregulation of the selected genes was associated with overall survival. The results obtained indicate that these genes could be employed as predictive biomarkers or potential targets to improve the effectiveness of CDDP treatment in lung cancer patients.

## 1 Introduction

Lung cancer is one of the leading causes of cancer-related mortality worldwide, according to GLOBOCAN (1). Lung cancer is divided into small cell lung carcinoma (SCLC) and non-small cell lung carcinoma (NSCLC). The latter group accounts for 85% of diagnosed cases and adenocarcinoma is the most frequent histologic type (2). Despite the great efforts that have been made in lung cancer diagnosis, most lung cancer patients are diagnosed at advanced stages, where metastasis is already present, limiting the treatment options to targeted or conventional therapy (2). According to the National Comprehensive Cancer Network guidelines for lung cancer treatment, patients whose tumors harbor *EGFR*-activating mutations must be treated with targeted therapy using tyrosine kinase inhibitors (TKIs) (3). However, not all patients benefit from treatment with TKIs, since some patients do not present favorable responses at treatment onset, or responders eventually acquire resistance. In both cases, or in patients not harboring *EGFR* mutant tumors, administration of cisplatin (CDDP) is the standard care treatment (3, 4). CDDP mainly affects cells that show high proliferation rates by forming adducts at the N-7 position of purines, causing DNA lesions that inhibit cell proliferation and induce cell death (5). However, the effectiveness of CDDP treatment in lung cancer remains limited due to the acquisition of therapy resistance (6). Although the mechanisms of CDDP-acquired resistance have been extensively studied in distinct types of cancer (7), little is known about the early molecular alterations that could be related to CDDP intrinsic resistance.

Recent reports demonstrate that non-genetic processes play a critical role in the development of resistance against different anti-tumoral agents (8, 9). During this process, a sub-population of cells known as drug-tolerant persister (DTP) cells survive the initial exposure to chemotherapy or targeted therapy. DTP cells are characterized by limited proliferation, altered metabolism associated with dormancy, activation of chromatin-remodeling enzymes, and activation of less error-prone DNA polymerases (10–12). These DTP cells represent a reservoir for the emergence of clones showing irreversible genetic drug resistance. Initial studies from Sharma et al. demonstrated that *EGFR*-mutant lung adenocarcinoma TKI-DTP cells exhibited altered chromatin remodeling and overexpression of insulin-like growth factor receptor 1 (IGF-1R). Thus, targeting IGF-1R and chromatin-modifying enzymes allowed this resistance to be overcome, eliminating DTP cells (13). Hangauer et al. demonstrated that DTP cells of breast cancer cell lines upregulated their antioxidant metabolism, rendering these cells susceptible to knockdown or pharmacological inhibition of the enzyme glutathione peroxidase-4 (14). Recently, research in osteosarcoma cell lines revealed that CDDP-DTP cells reprogram their transcriptome by altering the expression of genes of the MAPK, TGF-β, and NF-κB pathways, suggesting promising targets to ablate these cells (15). Therefore, DTP cells represent an excellent target to overcome resistance to antitumoral agents. Currently, most *in vitro* studies using DTP cells are focused on molecular changes elicited after several weeks of drug exposure. However, the key mechanisms governing the earliest stages of the emergence of CDDP-DTP cells in lung cancer remain to be understood.

In the present work, lung adenocarcinoma cell lines were single exposed to CDDP to analyze the earliest molecular changes associated with the emergence of CDDP-DTP cells. After 24 h of CDDP exposure, residual DTP cells exhibited transcriptional changes, which allowed them to survive the initial insult of the cytotoxic treatment. We employed RNA-seq to examine the transcriptional changes of early CDDP-DTP cells and identify key genes associated with this event. Interestingly, this early DTP state was controlled by a network of protein-coding genes participating in chromatin remodeling, metabolism of lipid kinases, and cell proliferation. Clinical validation using public datasets revealed that the overexpression or underexpression of these hub genes is associated with better or worse overall survival (OS) of lung cancer patients treated with CDDP. Among these genes, overexpression of the Suppressor of Cytokine Signaling 1 (SOCS1) was notably associated with poor survival of lung cancer patients. Furthermore, high expression of SOCS1 was associated with resistance to CDDP in the lung cancer cell lines employed. Further studies are needed to deepen the understanding of the role of SOCS1 in the chemoresistance process. These hub genes could represent promising predictive biomarkers or potential therapeutic targets, leading to improved efficacy of CDDP treatment and clinical outcome for lung cancer patients.

## 2 Materials and methods

### 2.1 Cell lines and culture conditions

The A549, H1299, and H1573 cell lines acquired from the ATCC (Manassas, VA, USA), and the 3B1A cell line, previously established by our group, were studied (16). All cell lines were obtained from treatment-free patients with lung adenocarcinoma, and do not harbor mutations in the *EGFR* gene. All cell lines were cultured in RPMI-1640 medium supplemented with 10% heat-inactivated FBS and 1% antibiotics (complete media) and incubated in a humidified atmosphere at 37°C with 5% CO_2_. Cisplatin (CDDP) was purchased from Sigma-Aldrich, cat. P4394 (St. Louis, MO, USA) and dissolved in isotonic saline solution. Dilutions were performed using complete media prior to each experiment.

### 2.2 Dose–response curves of CDDP in lung adenocarcinoma cell lines

After cell cultures reached 80% confluence, cells were harvested by enzymatic treatment and their viability always exceeded 95%. Cells were seeded in 96-well plates: A549 and H1299 cell lines at 1.2x10^4^ cells and H1573 and 3B1A cell lines at 5x10^4^. Cells were cultured overnight to allow cell attachment to plastic. Then, cell cultures were exposed to serial dilutions of CDDP ranging from 5 to 160 μM for 24 h. The plasmatic concentration of CDDP reported in lung cancer patients, which is 10 – 30 µM, was included in the concentration range tested in cell lines (17, 18). After treatment, 3-(4,5-dimethylthiazol-2-yl)-2,5-diphenyltetrazolium bromide (MTT) (Trevigen, Gaithersburg, MD, USA, cat. 4890-25K) was added to each well and the plate was incubated for 4 h at 37°C. Then, supernatants were discarded and resulting formazan crystals were dissolved with 150 μL of DMSO. Absorbance was recorded at 560 nm in a Multiskan Ascent plate reader (Thermo Fischer Scientific, Waltham, MA, USA). The percentage of cytotoxicity was calculated considering the readout of untreated cells as 0% cytotoxicity. From the results, dose–response curves were obtained. Three independent experiments, each one in triplicate were performed.

### 2.3 Annexin-V/PI assay

To quantify the percentage of viable, early, and late apoptotic/necrotic cells after CDDP exposure, the Annexin-V/PI assay was employed. A549, H1299 (7x10^5^ cells for both cell lines), H1573, and 3B1A (3x10^6^ cells for both cell lines) cells were seeded in T-25 flasks and cultured overnight to allow cell attachment. Next day, CDDP was diluted in fresh complete media and added to cell cultures. After treatment, floating cells were collected, adherent cells were detached using trypsin, and both tubes were mixed. For a rigorous examination of cell death events, cells were divided in two fractions. One fraction was employed for performing the Annexin-V/PI assay and the other fraction for caspase-3/7 activity measurement (see below). For the Annexin-V/PI assay, cells were washed with ice-cold Ca^2+^Mg^2+^-free PBS and rinsed in binding buffer. A total of 2-3x10^5^ cells were stained with FITC Annexin-V/PI, following instructions of the FITC Annexin V Apoptosis Detection Kit II (BD Pharmingen, San Diego, CA, USA, cat. 556570). A total of 15,000 events were immediately acquired in the FACS Canto II flow cytometer (Becton Dickinson, Franklin Lakes, NJ, USA). Since cell death is a time-dependent process, events were recorded at different times. However, only results from 24 h of exposure are shown. Two independent experiments were performed in triplicate. The percentages of viable, early, and late apoptotic/necrotic cells were calculated using the FlowJo (v10) software (Ashland, OR, USA).

### 2.4 LDH assay

To confirm the cytotoxic effect elicited by CDDP, lactate dehydrogenase (LDH) released in the culture media was measured using a cytotoxicity detection kit (Promega, WI, USA, cat. J2380). Cell cultures were seeded in 96-well plates under the same experimental conditions previously mentioned. After CDDP exposure, 50 μL of supernatants were mixed with 150 μL of the reaction mixture. Additionally, a positive control (cells treated with 1% Triton X-100) was included following instructions of the manufacturer. The plate, protected from light, was incubated at room temperature (RT) for 30 min and absorbance was immediately recorded at 490 nm using the Multiskan Ascent plate reader. The percentage of released LDH was calculated according to the equation previously reported (19). Three independent experiments, each one in triplicate were performed.

### 2.5 Caspase-3/7 activity assay

To verify whether CDDP-induced cell death was mediated by apoptosis, we measured the activity of caspase-3/7 in cell cultures after CDDP exposure. The Caspase-3/7 fluorometric assay kit (Promega, WI, USA, cat. G8981) was employed following instructions of the manufacturer. As previously indicated, cells from the remaining fraction of the Annexin-V/PI assay were centrifuged and pellets were rinsed in ice-cold lysis buffer and incubated on ice for 10 min. An amount of 50 μL of each extract was mixed with 50 μL of reaction buffer containing the DEVD-AFC substrate in a black 96-well plate. For each cell line, negative (untreated cells), positive (only floating dead cells), and blank controls were included. Once the substrate was added, the plate was incubated at 37°C for 2 h and fluorescence was measured in the Fluoroskan Ascent FL microplate reader (Thermo Fischer Scientific, Waltham, MA, USA) using the 390 and 485 nm excitation/emission filters. Caspase-3/7 activity was normalized per mg of protein. Thereby, total protein was quantified from each cell extract using the Micro BCA protein assay kit (Thermo Fischer Scientific, cat. 23235) in the Multiskan Ascent plate reader at 562 nm. The results are reported as the fold-change in the activity of caspase-3/7 with respect to untreated cells. Two independent experiments, each one in triplicate were performed.

### 2.6 Cell cycle analysis in residual cells

To investigate cell cycle alterations, cell lines were cultured in T-25 flasks under the same experimental conditions previously mentioned. After treatment, cells were collected, washed in PBS, fixed in 70% (v/v) cold ethanol, and stored at -20°C for at least 24 h. Then, cells were permeabilized with PBS containing 0.1% (v/v) Triton X-100 and treated with RNase A (30 μg/mL) (Thermo Fischer Scientific, Waltham, MA, USA, cat. EN0531) to avoid non-specific RNA staining. Finally, propidium iodide (PI) (Invitrogen, Waltham, MA, USA) was added for DNA staining, and the cells were incubated for 30 min at RT. After incubation, the DNA content was measured using the FACS Canto II flow cytometer. The first step in the analysis was to gate the population of singlet cells using an FSC-A/FSC-H dot plot. Then, a new gate for residual viable cells (CDDP-DTP cells) was set, from which a PI-A *vs*. FSC-A dot plot was obtained. Histograms were constructed to quantify the proportion of cells in each phase of cell cycle using FlowJo (v10) software. For this quantification, a total of 10,000 events were acquired from the gate of viable cells. Three independent experiments were performed in triplicate.

### 2.7 RNA-seq

To identify the genes related to the CDDP intrinsic resistance of lung adenocarcinoma cell lines, we sequenced the transcriptome of CDDP-DTP and untreated cells and performed differential expression analysis using bulk RNA sequencing. Cell lines were exposed to CDDP under the same experimental conditions previously mentioned. After treatment, to guarantee the collection of only adherent (CDDP-DTP) cells, we discarded the supernatants containing dead cells and washed the cell cultures several times, using fresh complete media, until floating/dead cells were not visualized in the microscope. Then, adherent cells were harvested by trypsinization, and cell viability was always higher than 95%. Total RNA was isolated from CDDP-DTPs and untreated cells (control) using the RNAeasy kit (Qiagen, Germany, cat. 74004). The quality of RNA was evaluated using the Agilent Bioanalyzer 2100 system (Agilent, Santa Clara, CA, USA) and always exceeded RIN > 8. For library preparation, we employed 2 µg of RNA per sample as input using the TruSeq RNA Sample Prep Kit v2 (Illumina, San Diego, CA, USA) and following directions of the manufacturer. Polyadenylated RNA was isolated using magnetic beads with polydT. Libraries were sequenced on the NextSeq 500 platform (Ilumina, San Diego, CA, USA) at a depth of approximately 30 million reads and 2 x 75 bp paired-end reads were generated. We sequenced the libraries of three independent experiments in duplicate.

### 2.8 Bioinformatic analysis

The quality of sequenced raw reads was examined using the FastQC (20) (v0.11.9) and MultiQC (v1.6) software (21). Raw reads were trimmed to eliminate low quality sequences (PhredScore < 25) and the presence of Illumina adapters using Trimmomatic (v0.38) and Cutadapt (v2.7), respectively (22, 23). Cleaned reads were aligned and mapped to the human genome GRCh38 (release 95) using STAR (v2.7.3a) (24) and abundance estimation of aligned reads was quantified using RSEM (v1.3.1) (25). Then, genes were tested for differential expression using edgeR (v3.32.1) (26), preserving those genes with a mean of one count per million across samples. The library size was normalized using the Trimmed Mean of M-values (TMM) method, and data were fitted to a negative-binomial model to estimate gene dispersion (common, trended, and gene-wise). Differential expression analysis was performed and genes showing a False Discovery Rate (FDR) of < 0.01 and |log2 fold change| > 1 were considered as differentially expressed. For functional annotation of the transcriptome of CDDP-DTP, Gene Set Enrichment Analysis (GSEA), employing gene sets retrieved from the Molecular Signature database (MSigDB), were performed using the fGSEA (v1.16.0) package. Enriched pathways showing a *p*-adjusted value of < 0.05 were considered as significant.

To construct the gene association network, the list of differentially expressed genes was analyzed using the Search Tool for the Retrieval of Interacting Genes/Proteins (STRING) database with the default parameters. Network analysis was conducted using the igraph (v1.2.10) package from R. Self-loops, and node-redundant connections were discarded. Additionally, only empirical genes (i.e., those present in the original list) were kept. Topological analysis of the network included the calculation of centrality measures such as total degree, betweenness, closeness, and eigenvalue. To select the most influencing centrality measure, PCA analysis was performed. For obtaining highly connected subnetworks, community detection analysis was conducted and functional annotation of the resulting subnetworks was performed using clusterProfiler (v3.18.1) (27).

### 2.9 Real-time quantitative PCR

To validate the results obtained in the bioinformatic analysis of RNA-seq data of CDDP-DTP cells, RT-qPCR was performed in the lung adenocarcinoma cell lines. Briefly, cell lines cultured in T-25 flasks were exposed to CDDP under the experimental conditions indicated above. After treatment, adherent cells from control or CDDP-treated cell lines were collected for total RNA isolation using the PureLink RNA Minikit (Ambion, Austin, TX, USA, cat. 12183018A). cDNA was synthesized using the High Capacity cDNA Reverse Transcription Kit (Ambion, Austin, TX, USA, cat. 4368814). A set of TaqMan probes (Applied Biosystems, Thermo Fischer, USA) was employed to amplify: *SOCS1, GADD45A, HEXIM1, HBEGF, BUB1B, KIF18A, ERCC6L*, and *NR2F2*. The housekeeping gene *GAPDH* was employed as an endogenous control (**Supplementary Table 1**). Gene amplification was performed in a StepOne Real Time PCR System (Thermo Fischer Scientific, Waltham, MA, USA). Data were normalized to the expression of the housekeeping gene, relative expression was calculated by the 2^-ΔΔCT^ method, and the log2 value is shown. Two independent experiments, each one in triplicate, were performed.

### 2.10 Survival analysis

By using clinical public data from the lung adenocarcinoma (LUAD) project of The Cancer Genome Atlas (TCGA) database, we investigated the relevance of the hub genes associated with patients’ OS. Clinical and expression datasets were retrieved using the RTCGA (v1.20.0) and TCGA-biolinks (v2.18.0) packages (28). Clinical data were curated by removing duplicated samples and preserving those from patients treated with CDDP. Patients were categorized using the maximally selected rank statistics method (29) with respect to the expression of high central genes associated with better or worse OS of patients. A Cox-regression test was conducted and Kaplan–Meier survival curves were obtained for each gene with their respective *p*-value (long-rank test) using the survival (v3.2-11) (30) and survminer (v0.4.9) packages. Log rank *p*-values of <0.05 were considered as statistically significant.

### 2.11 Western blotting

The protein expression levels of SOCS1 were examined by Western blot (WB). Total protein extracts from cell under the aforementioned experimental conditions were obtained. For this, cellular extracts from control and CDDP-DTP cells were obtained using NaCl (150 mM), Tris-HCl (50 mM), and Triton X-100 (1%) lysis buffer. Total protein was quantified using the MicroBCA Protein Assay Kit. A total of 25-40 µg of protein was loaded and resolved in 12% SDS-PAGE and subsequently transferred onto a nitrocellulose membrane using the Trans-Blot Turbo Transfer System (Bio-Rad, USA). Then, membranes were incubated in 2% BSA in PBS for 30 min to block non-specific binding sites. The primary antibody for SOCS1 (dilution 1:150, Abcam, UK, cat. ab137384) or actin β (dilution 1:10, 000, Sigma-Aldrich, Burlington, MA, USA, cat A1978-200UL) were applied to membranes, respectively, and incubated at 4°C overnight. After washing, membranes were incubated with corresponding biotinylated species-specific secondary antibodies, anti-rabbit or anti-mouse, (dilution 1:1,000, Invitrogen, USA, cat. 65-6140 and 31803) for 1 h at RT. Membranes were washed and incubated with the ABC complex at 1:300 for 30 min (Vector Laboratories, CA, USA, cat. PK-6100) and protein bands were visualized using the ECL kit (Cytiva, USA, cat. RPN2235) using the BioRad Universal Hood II Gel Doc System (BioRad, Hercules, CA, USA). Images were acquired using the Quantity One (v4.6) software (BioRad, Hercules, CA, USA). Band density was analyzed using the ImageJ software (v1.53), and results were normalized with respect to the signal of actin β. The results are expressed as the fold-change in intensity with respect to control cells. Three independent experiments were performed for each cell line studied.

### 2.12 SOCS1 localization in CDDP tolerant-persister cells

To detect the cellular localization of SOCS1 in untreated and residual cells after CDDP treatment, indirect immunofluorescence (IF) staining was performed. Lung adenocarcinoma cell lines were cultured in four-chamber slides (Lab-Tek, USA), washed, and fixed with ethanol. Then, cells were washed and treated for 30 min with blocking solution to avoid non-specific binding. The slides were incubated with SOCS1 antibody (dilution 1:150, Abcam, UK, cat. ab137384) for 2 h in a humidified atmosphere at 37°C. After washing, the slides were incubated with Alexa Fluor 488-conjugated secondary antibody (dilution 1:250, Invitrogen, USA, cat. A11070) at 37°C for 90 min. Finally, the cells were incubated with DAPI (dilution 1:150, Sigma-Aldrich, St. Louis, MO, USA, cat. 62248) for nuclear staining during 15 min. The slides were mounted with Vectashield (Vector Laboratories, CA, USA, cat. H1000) and micrographs were acquired using the EVOS FL microscope (Thermo Fisher Scientific, Waltham, MA, USA). Two independent experiments were performed.

### 2.13 SOCS1 knockdown

Since the 3B1A cell line has high SOCS1 expression previous to treatment, we studied the effect of SOCS1 knockdown in the sensitivity to CDDP. Briefly, cells were seeded in 48-well plates (1x10^5^ cells per well) and cultured overnight to allow attachment. Then, cells were washed with RPMI without FBS, and maintained in serum-free media for 4 h. Knockdown was performed with TriFECTa RNAi Kit in OptiMEM medium (Thermo Fisher, Waltham, MA, USA, cat. 31985), according to the manufacturer’s instructions. Cells were incubated with 3μL of lipofectamine 3000, (Invitrogen, USA, cat. L3000-015) and mixes of DsiRNAs SOCS1 (IDT, USA, HS.Ri.SOCS1.13.1-3) or HPRT1 (IDT, USA, HPRT1-S1) at final concentrations of 10nM for 48 h, at which maximum knockdown was obtained. Controls employed were lipofectamine alone (Mock), negative control (DsiRNA nontargeting human genes), and DsiRNA for an unrelated gene (HPRT1). DsiRNA sequences for knockdown are provided in **Supplementary Table 2**.

Knockdown efficiency was determined by RT-qPCR. At 24h of knockdown, cells were single exposed to CDDP, and cell viability was evaluated by dose response curves employing MTT, as described above.

### 2.14 Statistical analysis

Data were expressed as mean ± SD. For comparison, experimental and control groups were analyzed by Student’s *t-*test. For comparison among groups, we used the ANOVA test, and Tukey’s *post hoc* test. Unless otherwise specified, statistical analysis was performed using Prism 8 (GraphPad Software, La Jolla, CA, USA). A *p*-value of less than 0.05 was considered as statistically significant.

## 3 Results

### 3.1 Lung cancer cell lines show different sensitivity to CDDP

**Figure 1:**
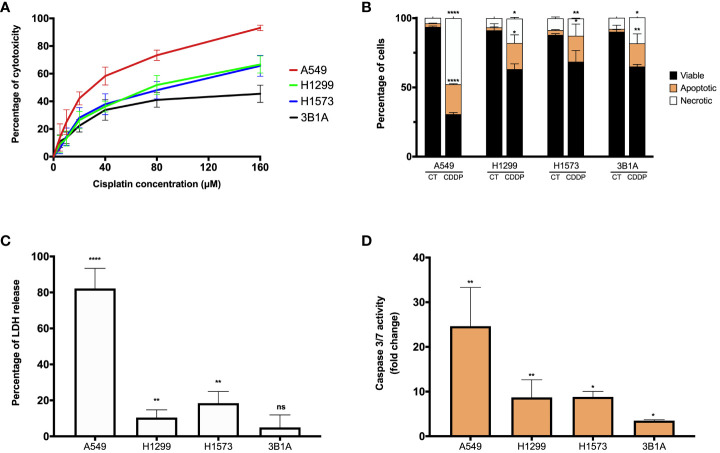
CDDP induces a cytotoxic effect in lung adenocarcinoma cell lines. **(A)** Dose – response curves of CDDP cytotoxicity in adenocarcinoma cell lines are shown. Three independent experiments were performed in triplicate. **(B)** The percentages of viable, early, and late apoptotic/necrotic cells are shown after 24 h of exposure to CDDP. Two independent experiments were performed in triplicate. **(C)** The cytotoxic effect of CDDP was confirmed by the release of LDH by necrotic cells in cell culture supernatants after 24 h of CDDP exposure. Three independent experiments were performed in triplicate. **(D)** Fold change of caspase-3/7 activity is expressed comparing the Relative Fluorescence Units of CDDP-treated and untreated cells from each cell line. Two independent experiments were performed in triplicate. Data are shown as the mean ± SD. The significant difference between control and treated cells is indicated with asterisks (* *p* < 0.05, ** *p* < 0.01, **** *p* < 0.0001, ns, not significant).

CDDP is an antitumoral agent employed as a standard-care treatment for cancer, owing to its cytotoxic activity. In our study, we evaluated the cytotoxic effect of CDDP in four *EGFR*-WT lung adenocarcinoma cell lines. In the A549 cell line, we observed a high proportion of cell death at the higher concentration of CDDP employed, which induced approximately 90% cytotoxicity (**Figure 1A**; **Supplementary Figure 1A**). The H1299 and H1573 cell lines showed a mild sensitivity, since the CDDP induced 60% cytotoxicity (**Figure 1A**). After exposure to CDDP, both cell lines presented a lower proportion of dead cells compared to A549 cells (**Supplementary Figure 1A**). Conversely, the 3B1A cell line showed the highest resistance to CDDP, since the drug induced 40% cytotoxicity (**Figure 1A**). In this cell line, a scarce proportion of dead cells was observed (**Supplementary Figure 1A**). These results indicate that each lung adenocarcinoma cell line shows a specific sensitivity to CDDP.

To examine whether CDDP-induced cell death was mediated by apoptosis, the percentages of viable, early, and late apoptotic/necrotic cells were measured using the Annexin-V/PI assay at the final time of CDDP exposure (**Supplementary Figure 1B**). In A549 cells, CDDP induced a significant increase (*p* < 0.0001) of 8.5-fold and 12-fold in the proportion of apoptotic and necrotic cells, respectively, compared to control cells (**Figure 1B**). In the H1299, H1573, and 3B1A cells, CDDP induced a similar effect, as apoptotic and necrotic cells significantly (*p* < 0.05) increased 9 – 10-fold and 2 – 2.5-fold, respectively (**Figure 1B**). These results are in line with those obtained in the MTT assay.

To confirm the cytotoxic effect induced by CDDP, we evaluated the release of the enzyme LDH as a biochemical marker of cell death. In the A549 cell line, the most sensitive to CDDP, a significant increase of 80% (*p* < 0.0001) in the release of LDH was detected. In contrast, in H1299 and H1573 cell lines CDDP exposure caused a release of approximately 10 – 20% (*p* < 0.005) of the enzyme. The 3B1A cell line, showing resistance to CDDP, caused a marginal release of LDH of less than 5% (**Figure 1C**). To corroborate the apoptotic cell death induced by CDDP, we measured the activity of caspases 3/7. In the A549 cell line, CDDP exposure significantly increased (25-fold, *p* < 0.005) the activity of these caspases. H1299 and H1573 cells, which showed mild sensitivity to CDDP, a significant increase of eight-fold (*p* < 0.05) in activity of caspases 3/7 was detected. In 3B1A cells, an increase of five-fold (*p* < 0.05) in the activity of these executioner caspases was detected. Comparisons were performed with respect to the corresponding control cells of each cell line (**Figure 1D**).

In summary, all these results indicate that CDDP has a different cytotoxic effect among lung adenocarcinoma cell lines. It is important to highlight that in all cell lines after CDDP exposure we observed a fraction of viable cells which could be related to DTP cells.

### 3.2 Identification of cell cycle alterations in residual cells

Previous studies report that DTP cells exhibit limited proliferation related to dormancy (8, 31). For this reason, we decided to evaluate alterations in the cell cycle phases in CDDP-residual cells. In the A549 cell line, treatment induced a significant reduction of cells in the S and G2/M phases and a significant increase of 1.4-fold (*p* < 0.0001) in the proportion of cells in the G0/G1 phase. In H1299 cells, CDDP exposure reduced the number of cells in the G0/G1 and G2/M phases. Surprisingly, CDDP induced a significant increase in the number of cells in the S-phase (3.5-fold, *p* < 0.001). After exposure, H1573 showed a slightly increase in the number of cells in the S and G2/M phases with a concomitant decrease of cells in the G0/G1 phase. In 3B1A cells, CDDP caused accumulation of cells in G2/M, since a two-fold (*p* < 0.005) increase of cells in this phase was observed (**Supplementary Figures 2A–C**). These results suggest that CDDP-residual cells show cell cycle alterations related to the DTP state.

### 3.3 Transcriptomic profile of CDDP-DTP cells

**Figure 2:**
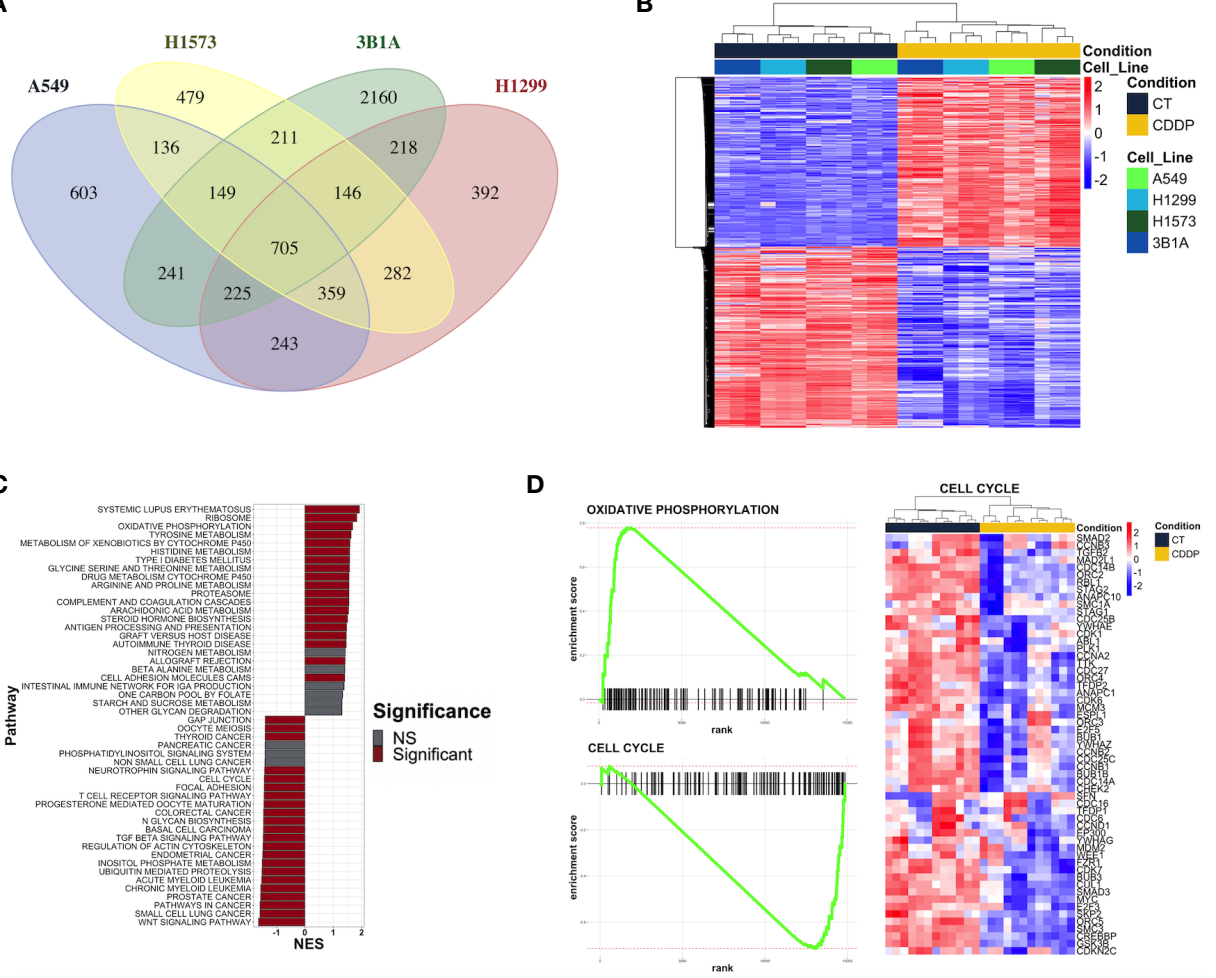
Transcriptional alterations in the early lung adenocarcinoma CDDP-DTPs cells. **(A)** Venn diagram showing the common differentially expressed genes (|log_2_ fold change| ≥ 1 and *p*-adjusted value < 0.01) regulated in CDDP-DTPs cells from the four cell lines. **(B)** Heatmap showing the unsupervised hierarchical clustering of control and CDDP-DTPs cells using the mean-centered log_2_ TMM-normalized counts of the common differentially expressed genes. Sample clustering was performed calculating the Euclidean distances and the single method. **(C)** Gene set enrichment analysis of the transcriptome of CDDP-DTPS cells using KEGG data sets retrieved from MSigDB. The normalized enrichment score (NES) is shown. **(D)** Enrichment plots of the most significant gene sets detected in the GSEA analysis. CT (control cells), CDDP (CDDP-treated cells), and NS (not significant).

We performed RNA-sequencing of poly-adenylated RNA from CDDP-DTP cells and control cells and tested for differentially expressed genes. Unsupervised reduction of dimensions revealed that the main source of variation in our datasets was the CDDP treatment, since control and drug-persister cells tended to form distinct clusters in the first component (PC1), indicating that CDDP-DTP cells showed a different transcriptional profile (**Supplementary Figure 3**). Additionally, a high degree of reproducibility in the biological replicates from both experimental groups in each dataset was found. Differential expression analysis between DTP and control cells found distinct proportions of differentially expressed genes (DEGs) (|logFC| > 1 and FDR < 0.01) for each cell line (**Supplementary Figure 4**). When all datasets were analyzed, CDDP-DTP cells from the four cell lines shared a total of 705 DEGs (343 upregulated and 362 downregulated), showing consistent expression across the persister cells of the four cell lines (**Figure 2A**). Interestingly, unsupervised hierarchical clustering analysis using the expression of these 705 shared DEGs, showed that CDDP-DTP cells tended to cluster according to the degree of sensitivity detected in previous assays (**Figure 2B**). Gene-set enrichment analyses showed that the transcriptome of CDDP-DTP cells is positively enriched in genes participating in pathways associated with oxidative phosphorylation, cytochrome P450-mediated detoxification of drugs, RNA metabolism and proteasome activity (**Figures 2C, D**), suggesting that these pathways are activated. Contrarywise, negatively enriched pathways were involved in the activation of MAPK, TGF-β, and WNT signaling, as well as the cell cycle and apoptosis (**Figures 2C, D**), suggesting that these pathways are repressed in CDDP-DTP cells. These results corroborate our previous observations of the cell cycle alterations after treatment indicating that CDDP-DTP cells disabled the expression of genes involved in the cell cycle control (**Figure 2D**; **Supplementary Figure 2**).

**Figure 3:**
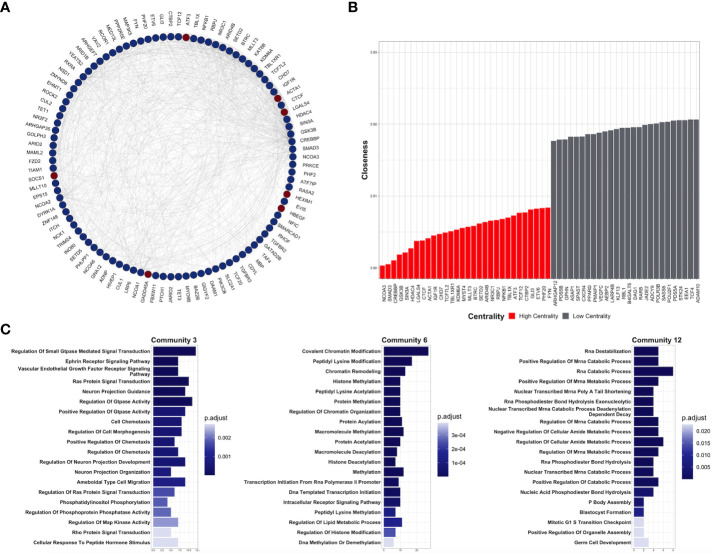
Key protein-coding genes in CDDP-DTPS cells. **(A)** Functional association network indicating the empirical interactions among the common differentially expressed genes of CDDP-DTP cells from the four cell lines. Node color depicts the expression of the genes, overexpressed (red) or underexpressed (blue). For clarity, genes detected as highly central are shown. **(B)** Topological analysis of the CDDP-DTP cell-associated gene signature network. The -log10 of closeness calculated during the topological analysis is shown. Genes ubicated in the upper-quartile were designated as “high centrality” (red) and those that did not meet the cut-off criteria as “low centrality” (gray). **(C)** Over-representation analysis of the most significant subnetworks, detected by community analysis, were performed using the Gene Ontology Biological Processes data set. Significant pathways (*p*-adjusted value < 0.05) are shown.

To understand the functional interaction among shared DEGs from CDDP-DTP cells, a protein–protein interaction (PPI) network was constructed using information from the STRING database (**Figure 3A**). Validation analysis showed that gene interactions of the resulting network were more significant (enrichment *p*-value < 10^-6^) than those obtained from a random selection of the same number of genes from the human genome. This result indicates that the CDDP-DTP-associated genes have a higher tendency to establish a network than would be expected by random chance. Further topological analyses of the constructed network showed that closeness was the centrality measure which had most information related to central nodes (**Supplementary Figure 5**). Using the -log10 of closeness value, 99 of 396 genes were identified as highly central (-log10 of closeness in the 75th percentile) (**Figure 3B**; **Supplementary Table 3**). These 99 genes were defined as hub genes. In support of these findings community detection analysis detected 25 subnetworks with at least five members. Almost 75% (74/99) of hub genes were distributed in the three largest subnetworks. Functional annotation of these subnetworks included activation of WNT, PI3KCA, and RAS GTPase pathways. Also, biological processes such as chromatin remodeling, post-translational modifications on histones, and regulation of mitosis were listed (**Figure 3C**).

Overall, these results indicate that CDDP-DTP cells have a specific transcriptomic profile in which genes related to pathways, such as drug detoxification, cell proliferation, and survival are altered. Additionally, the subset of CDDP-DTP-associated genes is strongly linked and participates in controlling the activation of pathways related to gene expression and cell proliferation.

### 3.4 RT-qPCR of hub genes of DTP cells

**Figure 4:**
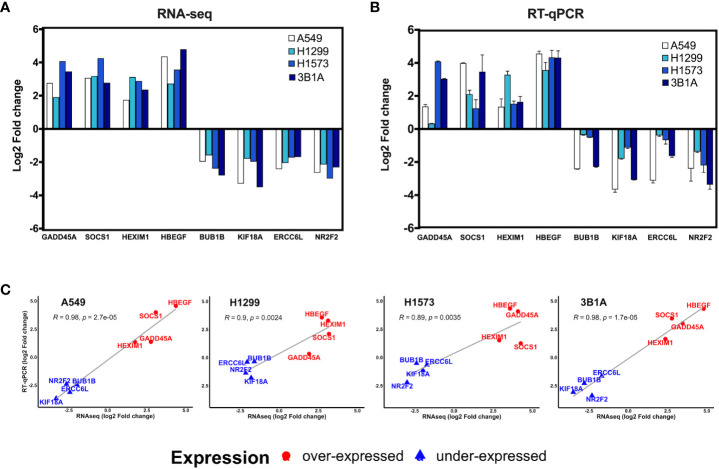
Validation of hub genes by RT-qPCR. Log2-fold change of the expression of CDDP-persister state hub genes from **(A)** RNA-seq or **(B)** RT-qPCR experiments. **(C)** Scatter plots depicting the high correlation between RNA-seq and RT-qPCR data from the four cell lines tested (Pearson’s correlation coefficient and *p* value are indicated).

We analyzed the expression of 8 of the 99 hub genes found in CDDP-DTP cells by RT-qPCR, using RNA samples independent from those employed for transcriptome sequencing. For this analysis we selected four overexpressed hub genes (*GADD45A, SOCS1, HEXIM1*, and *HBEGF*), and four underexpressed hub genes (*BUB1B, KIF18A, ERCC6L*, and *NR2F2*). We found that the expression of the selected hub genes was concordant between RNA-seq and RT-qPCR, showing a significant correlation in the four lines studied (**Figures 4A–C**).

### 3.5 Different expression of CDDP-DTP-associated hub genes between tumor and normal tissue

Data retrieved from the LUAD project of the TCGA database were used to analyze the clinical significance of the hub genes. First, we compared the expression of hub genes between tumor and normal adjacent tissue. Only the expression of *GADD45A* was significantly increased in tumor samples (*p* < 0.05) compared to normal tissue, which is in line with the overexpression we found in CDDP-DTP cells (**Figure 4**). In contrast, the expressions of *SOCS1, EPS15, GLI3, NR2F2*, and *RCOR1* were significantly decreased in tumor compared to normal tissue (*p* < 0.05) (**Supplementary Figure 6**).

### 3.6 Association of CDDP-DTP hub gene expression and overall survival of LUAD patients

**Figure 5:**
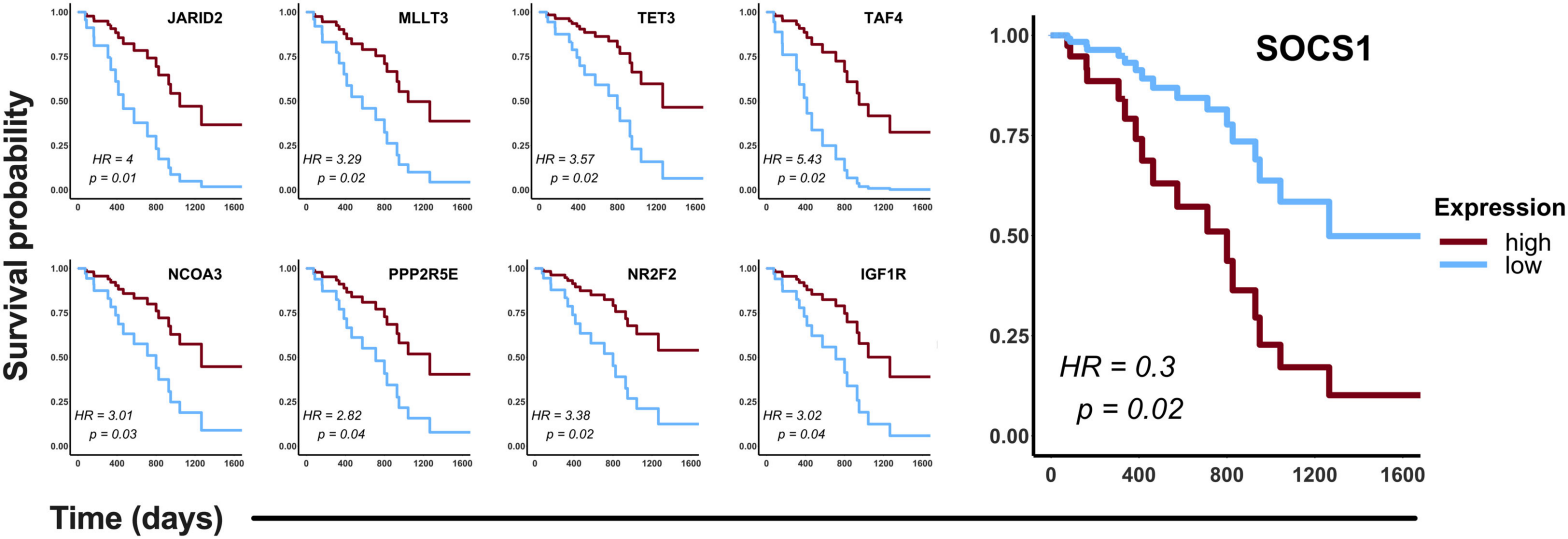
Clinical relevance of the hub genes detected in the CDDP-DTP cells in a cohort of CDDP-treated patients. Publicly available data of 87 CDDP-treated patients was retrieved from the LUAD project of the TCGA. Patients were categorized in two groups: high expression (red) and low expression (blue). Hub genes showing a significant (log-rank *p* < 0.05) association with OS of patients are shown. High expression of eight hub genes was associated with better OS, whereas only high expression of SOCS1 was associated with poor OS of CDDP-treated LUAD patients.

To validate *in silico* the clinical significance of the hub genes, the association of their expression with the OS of CDDP-treated patients was analyzed using data retrieved from the LUAD project of the TCGA database. Analyses were conducted in the cohort of patients (n= 87) treated with CDDP, and Cox regression testing showed that 9 of 99 hub genes reached statistical significance with the OS of patients (*p*-value < 0.05, log-rank test). Low expression of *JARID2, MLLT3, TET3, TAF4, NCOA3, PPP2R5E, NR2F2, and IGF1R*, and high expression of *SOCS1* was associated with poor OS of the CDDP-treated LUAD patients (**Figure 5**). Interestingly, we found the same dysregulation of these hub genes in our transcriptomic analysis (**Supplementary Table 3**), which suggest that these gene signature may be related to the presence of CDDP-DTP cells and CDDP-treatment resistance. Moreover, six of the nine (60%) clinically relevant genes were part of the subnetwork 6 (Chromatin remodeling), suggesting a relationship between this process and the response to CDDP in patients (**Supplementary Table 3**).

### 3.7 SOCS1 protein expression in lung adenocarcinoma cell lines with different sensitivity to CDDP

**Figure 6:**
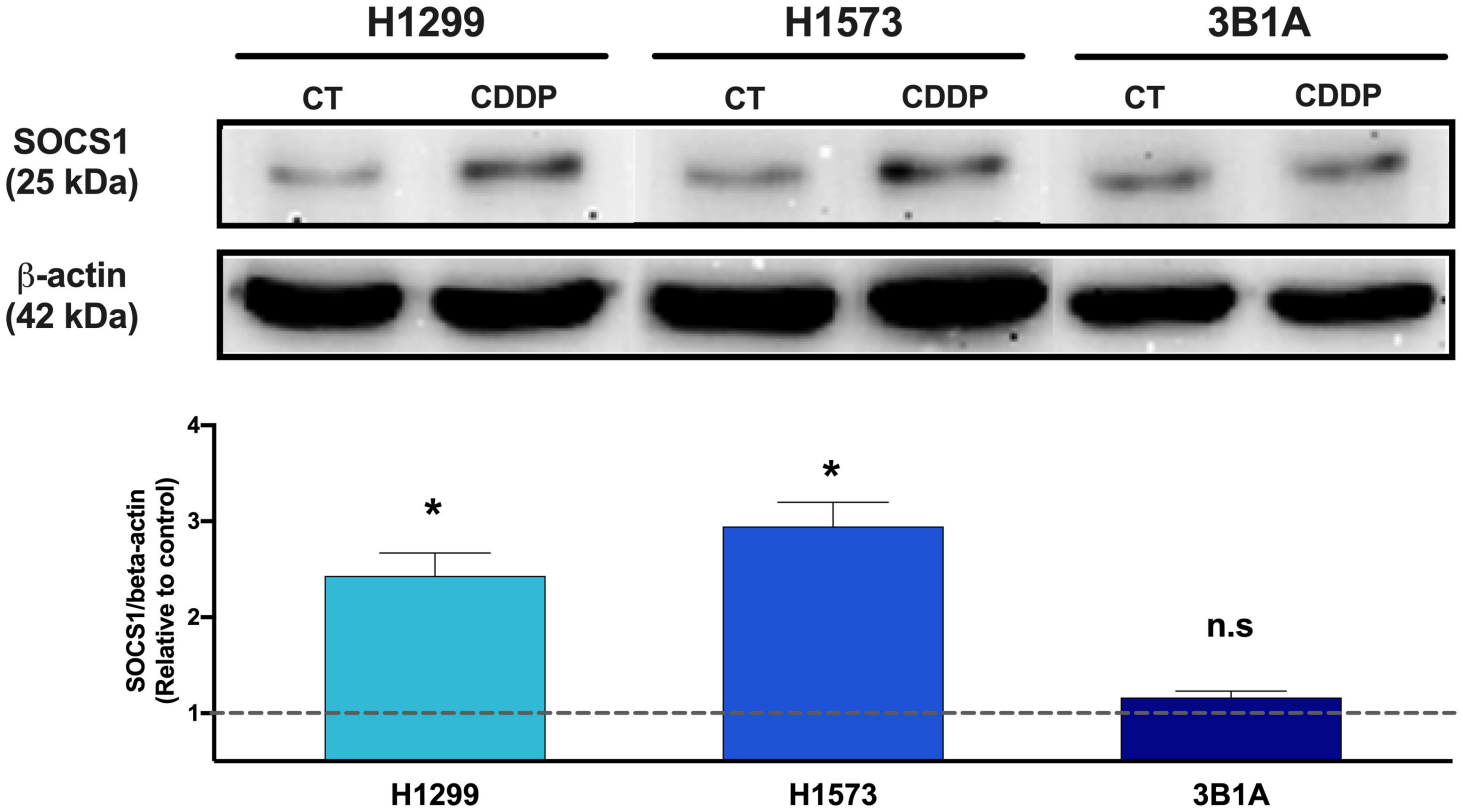
Comparison of SOCS1 expression in untreated cells (CT) and CDDP-DTPs (CDDP) from H1299, H1573, and 3B1A cell lines. Actin beta (42 kDa) was employed as an endogenous expression control. Fold change of SOCS1 expression respect to actin beta is indicated, comparing the relative units of CDDP-DTPs and control cells. Three independent experiments were performed. Data are shown as the mean ± SD. Significant difference between control and treated cells is indicated with asterisks (* *p* < 0.05, ns, not significant).

Since SOCS1 was found upregulated in CDDP-DTP cells and its overexpression correlated with poor OS in the cohort of LUAD patients treated with CDDP, we sought to analyze the protein expression of this molecule in the 3B1A cell line (resistant to CDDP), and in the H1573 and H1299 cell lines (mild sensitive to CDDP), in control and CDDP-DTP cells. Western blot (WB) analysis showed that the mildly sensitive cell lines H1299 and H1573 show low protein expression of SOCS1 before exposure, but these levels are significantly (*p* < 0.05) increased in CDDP-DTP cells, by 2.5-fold and 3-fold, respectively (**Figure 6**). In contrast, the 3B1A cell line has higher levels of SOCS1 before exposure to CDDP and displays no significant changes after CDDP exposure (**Figure 6**). This result supports the notion that high SOCS1 expression might be related to intrinsic resistance to CDDP.

### 3.8 Localization of SOCS1 in CDDP-DTP cells

**Figure 7:**
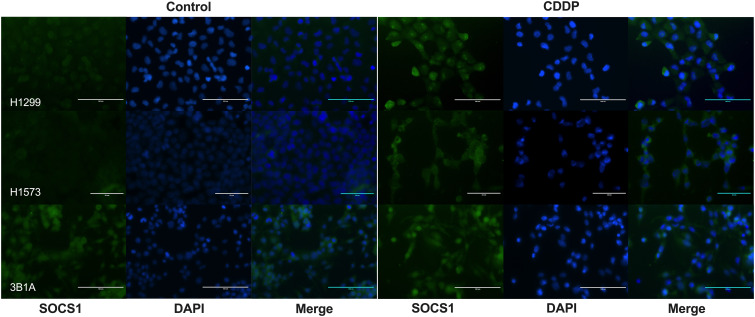
Cellular localization of SOCS1 in control and CDDP-DTPs (CDDP) in the lung adenocarcinoma cell lines. In H1299 and H1573 cells, a slight cytoplasmic staining of SOCS1 was observed. In both cell lines, CDDP exposure increased the cytoplasmic expression of SOCS1. In contrast, 3B1A cells expressed SOCS1 before treatment and remained unchanged after CDDP exposure. DAPI was employed for nuclear staining. Magnification X400 for H1299 and 3B1A cell lines and X600 for H1573 cell line.

To assess the localization of SOCS1 and corroborate the WB results, we performed IF on control and DTP cells. In H1299 and H1573 cells, a slight to no cytoplasmic expression of SOCS1 was observed prior to treatment. However, the fluorescence increased in CDDP-DTP cells. In comparison, 3B1A cells showed a higher signal of cytoplasmic fluorescence of SOCS1 before treatment that was maintained after CDDP exposure (**Figure 7**). These observations are consistent with the protein expression detected by WB.

### 3.9 Effect of SOCS1 knockdown in CDDP resistance

**Figure 8:**
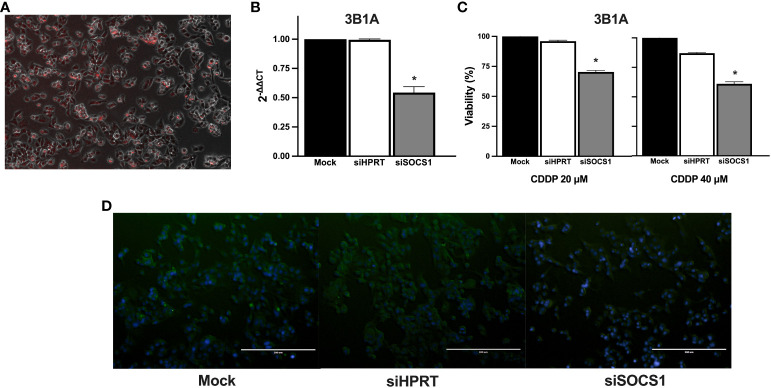
Effect of SOCS1 knockdown on the 3B1A cell line sensitivity to CDDP. **(A)** Micrograph showing above 80% of cells positive for TYE 563 control of Trifecta kit. **(B)** Diminishing of SOCS1 expression in DsiRNA-transfected 3B1A cells after 48h of transfection detected by RT-qPCR. **(C)** Changes in cell viability of mock, HPRT1 DsiRNA, and SOCS1 DsiRNA transfected cells, after 24h of CDDP exposure (20 μM and 40μM). **(D)** Comparison of SOCS1 expression in mock, HPRT1 DsiRNA, and SOCS1 DsiRNA transfected cells exposed to 40μM of CDDP. Magnification X400. * p < 0.001.

To further explore the role of SOCS1 in CDDP resistance, we performed DsiRNA-mediated knockdown in the CDDP-resistant 3B1A cell line and assessed its effect on CDDP cytotoxicity. Fluorescently labeled transfection control showed an efficiency of transfection above 80% (**Figure 8A**). RT-qPCR showed a 50% decrease in the mRNA expression of SOCS1, compared to mock control and cells transfected with an independent DsiRNA (HPRT1) (**Figure 8B**). The cell viability assay revealed that knockdown of SOCS1 reduced approximately 30 to 40% of cell viability of 3B1A cells, compared to control cells, at concentrations tested of 20 and 40μM of CDDP (**Figure 8C**).

Cells with SOCS1 knockdown decreased fluorescent compared to that of siHRTP and mock controls (**Figure 8D**).

Taken together, these results suggest that the differential expression of SOCS1 before and after treatment may be related to the phenomenon of intrinsic resistance as well as the differential sensitivity to CDDP previously observed in cell lines.

## 4 Discussion

Despite the development of new therapeutic strategies, CDDP remains as the standard care treatment for advanced lung tumors that do not harbor EGFR mutations or that show resistance to TKIs. However, not all lung cancer patients show a favorable response at treatment onset, and responders eventually acquire resistance to CDDP therapy. Previous reports indicate that during the development of acquired resistance to chemotherapy, a population of cells survives since the initial exposure. These cells are known as drug persister cells (10, 32). According to the drug persister model proposed by Lin and Shaw (33), these cells propagate and give rise to subclones that show acquired resistance to chemotherapy, leading to tumor relapse. Most reports indicate that DTP cells emerge after continuous exposure to antitumoral agents (10, 13, 34). Recent studies performed in osteosarcoma, melanoma, colorectal, and lung cancer cell lines showed that, after two weeks of drug exposure, DTP cells reprogram their transcriptome by altering key cellular pathways which favor their survival (8, 15, 35, 36). However, current research has not yet clarified the early molecular mechanisms underlying the intrinsic resistance against CDDP in drug persister cells in lung cancer. For this reason, we considered it necessary to investigate the transcriptomic alterations in CDDP-DTP cells after a single CDDP exposition and their impact on the emergence of drug resistant cell populations and subsequent tumor relapse. To our knowledge, this is the first study evaluating the earliest transcriptional changes associated with the drug-tolerant persister state after a unique exposure to CDDP in lung adenocarcinoma cell lines.

Bronte et al. (37) reported that one of the primary sources of variable sensitivity to chemotherapy in patients is the presence of pre-existing mutations in cancer driver genes. *TP53* is one of the most relevant genes controlling the cytotoxic effect induced by CDDP, since it activates the expression of genes involved in DNA damage, cell cycle, and cell death pathways (38, 39). Several reports have shown that mutations in *TP53* are associated with resistance to CDDP. Lisek et al. reported that mutant TP53 interacts and activates the antioxidant master regulator NRF2 in breast cancer cell lines, thus increasing the expression of antioxidant enzymes that neutralize CDDP inside the cell (40). In addition, in lung cancer cell lines, mutant TP53 induces the expression of micro-RNA-128-2, which inhibits apoptosis by downregulating the expression of the repressor E2F5, conferring resistance to CDDP (41). Lung cancer patients with TP53 mutations fare worse after CDDP treatment than those with wild-type (WT) *TP53* tumors (42, 43). Specific *TP53* mutations that cause structural alterations in TP53 protein are associated with poor OS in lung cancer patients treated with CDDP (42).

Results obtained in this study are consistent with these reports, since we found that lung adenocarcinoma cell lines harboring wild-type *EGFR* exhibited distinct sensitivity to CDDP that could be related to alterations in *TP53*. The A549 cell line, which expresses *TP53* WT, showed high sensitivity to CDDP compared to cell lines H1299 and H1573 that exhibit *TP53* alterations (null and c.743G>T, respectively). Consistent with the results obtained by Liu et al., the H1299 cell line became sensitive to CDDP after transfection with *TP53* (44). Interestingly, in a previous study using the TruSight Tumor 15 (Illumina, USA) (45) we found that the 3B1A cell line lacks mutations in *TP53* (Data not published). In the present study, we found that the 3B1A cell line showed the highest resistance to CDDP, suggesting that factors other than *TP53* mutations contribute to the observed differences in CDDP sensitivity among these cell lines.

In order to investigate other factors that may be participating in the early drug persistence or tolerant state related to CDDP resistance, we sought to evaluate the interactions among DEGs associated with the DTP state. Reports indicate that following radiation or CDDP exposure, nasopharyngeal and osteosarcoma cell lines reprogram their transcriptomes to survive the insult as soon as 24 h (15, 46). Similarly, we found that CDDP-DTP cells differentially expressed a subset of genes that could be related to the early drug-tolerant persister state. Functional annotation of the transcriptomes revealed that CDDP-DTP cells activate pathways associated with oxidative phosphorylation, drug metabolism, enzymes of cytochrome P450, and proteasome activity. These results are in accordance with previous findings of models of CDDP-acquired resistance in which detoxifying enzymes that neutralize the oxidative species of the drug were detected (47).

Additionally, the activation of oxidative phosphorylation has been reported to be linked with CDDP resistance in ovarian cancer by stimulating the production of pro-inflammatory cytokines such as IL-6 and IL-8, which favor the expression of membrane pumps involved in the extrusion of CDDP (48). We found that CDDP-DTP cells inactivate pathways associated with cell proliferation, such as the canonical WNT, TGF-β signaling, and cell cycle pathways. These results are in line with previous studies showing that at advanced exposure times, a population of DTP cells lose the capacity to cycle (8, 31). Moreover, Krtinic et al. reported that ovarian cancer cells with decreased gene expression associated with proliferation exhibit CDDP resistance (49). Accordingly, we found alterations in the distribution of the cell cycle phases of CDDP-residual cells. A possible explanation for this finding is that CDDP-DTP cells might be delayed in the cell cycle due to the DNA damaged caused by CDDP, as previously mentioned. Recent evidence in osteosarcoma and lung adenocarcinoma cell lines shows that after CDDP exposure, a residual population of tumor cells survives because they are delayed in proliferation, since they require time to activate DNA repair mechanisms to alleviate the stress caused by CDDP (50, 51). Our findings suggest that CDDP-residual cells survive the cytotoxic insult by altering the distribution of cells in cell cycle phases and decreasing their proliferation rate, which is associated with the DTP state.

One of the main objectives of the present work was to identify a transcriptional signature related to the emergence of early CDDP-DTP cells in lung adenocarcinoma cell lines. Prior studies have reported the importance of genes participating in decreased cell proliferation, chromatin remodeling, and metabolic alterations associated with dormancy after prolonged exposure to antitumoral drugs in cell lines (31, 34, 35). Liau’s and Vinogradova’s groups demonstrated that persister cells upregulate histone demethylases, such as KDM5 or KMD6B as a survival strategy (34, 52). Glioblastoma DTP cells, in addition to upregulating histone demethylases, also decrease the expression of cell cycle genes as a strategy to survive the cytotoxic effect of kinase inhibitors (52). These pathways remain altered for prolonged exposure times, and, in our study, we found that these pathways are involved since early stages of the persister state to CDDP.

The use of publicly available data bases such as the TCGA has facilitated progress in the study of the different cancer types. The analysis of DEGs between tumor and normal tissue has enabled the proposal of molecular markers for the diagnosis and prognosis of treatment response, especially in lung cancer. Comparing the expression of the CDDP-DTP-associated genes between tumor and normal adjacent tissue, and its clinical relevance, we found that six (*GADD45A, SOCS1, EPS15, GLI3, NR2F2*, and *RCOR1*) of the hub genes have significant differences. Interestingly, according to the data analyzed, the expression of *SOCS1* is lower in the tumor tissue than in normal adjacent tissue.

By analyzing the clinical relevance of the CDDP-DTP-associated hub genes with the five-year OS of CDDP-treated patients from the LUAD cohort of the TCGA, we found a positive correlation with the expression of eight of these hub genes (*JARID2, MLLT3, TET3, TAF4, NCOA3, PPP2R5E, NR2F2,* and *IGF1R*). With respect to the findings of SOCS1 in the OS of patients, which appear to be contrary to those reported in tumor tissue, overexpression of *SOCS1* correlates with poor OS of the CDDP-treated LUAD patients. This may be due to *SOCS1* expression could be related to the abundancy of tumor cells that pretreatment display the DTP state. These cells would be able to survive CDDP exposure and expand to favor treatment resistance and eventual tumor relapse. Further studies in tumor samples and liquid biopsies evaluating the expression of the proposed genes at treatment onset, with treatment, and incorporating results of Response Evaluation Criteria in Solid Tumors (RECIST) are required to confirm their biological relevance in lung adenocarcinoma patients.

Since overexpression of SOCS1 was associated with poor OS of CDDP-treated LUAD patients, which may suggest that it plays an important role in resistance to treatment, we wanted to evaluate if SOCS1 expression was associated with the different sensitivity to CDDP observed among cell lines. SOCS1 belongs to a family of eight intracellular proteins that negatively regulate the signaling induced by pro-inflammatory cytokines via JAK/STAT, TLR, and NF-κB activation (53). Alterations in SOCS1 activity are implicated in autoimmune diseases such as psoriasis, systemic lupus erythematosus, recurrent uveitis, and cancer (53). In this regard, the dichotomic participation of SOCS1 in cancer has been extensively discussed since, depending on the tumor stage, it displays both tumor-promoting or antitumor activities (54, 55). However, its role in chemotherapy resistance is not well understood. Our results suggest a direct relationship between the level of SOCS1 expression and the resistance of cell lines to CDDP. The 3B1A cell line, derived from an untreated patient, showed the highest resistance to CDDP and the highest expression of SOCS1, suggesting the presence of CDDP-DTP cells, which may emerge alongside tumor development. In comparison, H1299 and H1573 cell lines showed mild sensitivity to CDDP and lower SOCS1 expression; this expression augmented in residual cells. This suggests the existence of tumor cells that survive the initial exposure to CDDP and overexpress *SOCS1* in response to the insult as another possible source of CDDP-DTP cells. It has been reported that SOCS1 interacts with proteins of the DNA damage response such as ATM and ATR (56). In this setting, CDDP-DTP cells could alleviate the stress in DNA caused by CDDP by increasing the expression of SOCS1 at the transcript and protein levels. These findings suggest the role of SOCS1 in the intrinsic resistance against CDDP of lung cancer cell lines. However, further basic and clinical studies are needed to examine SOCS1 alterations and to precisely identify its molecular mechanism after CDDP exposure in lung adenocarcinoma and its relationship with treatment efficacy.

In accordance with previous reports, we found that SOCS1 was mainly localized in the cytoplasm, where it can interact with STAT family members, such as STAT3 and STAT1 (57). These results may be explained by the fact that STAT1-STAT3 heterodimer displays antitumor effects by regulating the transcription of genes participating in cell death (58).

To further confirm the role of SOCS1 in CDDP resistance, we transfected the 3B1A cell line with a DsiRNA against SOCS1. The increase in cell death of transfected cells supports the proposal that targeting of SOCS1 expression sensitizes a previously resistant cell line against CDDP.

In conclusion, our work provided a comprehensive network of the genes participating in the early stages of CDDP-DTP cells. The transcriptional changes associated with this state activated key cellular processes regulated by the interaction of critical protein-coding genes. We suggest that the genes in this signature display an important functional and clinical relevance for lung adenocarcinoma patients before CDDP treatment. This gene signature could represent a promising alternative to improve the outcome of lung cancer patients. Importantly, SOCS1 could be used as a response biomarker of CDDP treatment and as a potential target to overcome resistance to this standard chemotherapeutic.

## Data availability statement

The datasets presented in this study can be found in online repositories. The names of the repository/repositories and accession number(s) can be found in the article/**Supplementary Material**.

## Ethics statement

Ethical approval was not required for the studies on humans in accordance with the local legislation and institutional requirements because only commercially available established cell lines were used.

## Author contributions

Conceptualization and design, RC-D, DA-C, and JL-G. Methodology, RC-D, DA-C, MP-M, MS-N, LI-V, MG-V, JJB-L, and SA-R. Software, bioinformatics and statistical analyses, RC-D, MP-M, SA-R, and AM-T. Bioinformatic clinical validation, RC-D, MP-M, and AM-T. Writing—original draft preparation, RC-D, DA-C, and JL-G. Writing—review and editing, RC-D, DA-C, JJB-L, and JL-G. All authors contributed to the article and approved the submitted version.
