# Supplementary material for: Transcriptional signature of early cisplatin drug-tolerant persister cells in lung adenocarcinoma
Source: Front Oncol. 2023 Oct 17;13:1208403. doi: 10.3389/fonc.2023.1208403 (PMC10616253; doi:10.3389/fonc.2023.1208403)
Supplement: Supplementary file 1 [file DataSheet_1.docx]

Supplementary Material

Transcriptional signature of early cisplatin drug-tolerant persister cells in lung adenocarcinoma

**Rodolfo Chavez-Dominguez^1,2†,^ Dolores Aguilar-Cazares^1†^, Mario Perez-Medina^1,3†^ , Santiago Avila-Rios^4^ , Maribel Soto-Nava^4^ , Alfonso Mendez-Tenorio^5^ , Lorenzo Islas-Vazquez^6^ , Jesus J Benito-Lopez^1,2^ Miriam Galicia-Velasco^1^, and Jose S Lopez-Gonzalez^1*^**

1. Instituto Nacional de Enfermedades Respiratorias, Departamento de Enfermedades Cronico-Degenerativas, Laboratorio de Cancer Pulmonar, C.P 14080, Ciudad de Mexico, Mexico
2. Posgrado en Ciencias Biologicas, Unidad de Posgrado, Edificio D, 1° Piso, Circuito de Posgrados, Ciudad Universitaria, Coyoacan, C.P. 04510, Ciudad de Mexico, Mexico.
3. Escuela Nacional de Ciencias Biologicas, Instituto Politecnico Nacional, Ciudad de Mexico, Mexico.
4. Instituto Nacional de Enfermedades Respiratorias, Centro de Investigacion de Enfermedades Infecciosas, C.P 14080, Ciudad de Mexico, Mexico.
5. Laboratorio de Biotecnologia y Bioinformatica Genomica, Departamento de Bioquimica, Escuela Nacional de Ciencias Biologicas, Instituto Politecnico Nacional, CP 11340, Ciudad de Mexico, Mexico
6. Departamento de Inmunologia y Unidad de Investigacion, Instituto de Oftalmologia "Conde de Valenciana", Ciudad de Mexico 06800, Mexico.

**^†^** These authors share first authorship.

*** Correspondence:**Jose S. Lopez-Gonzalez
[slopezgonzalez@yahoo.com](mailto:slopezgonzalez@yahoo.com)

# Supplementary data

RNA-seq raw reads and count matrices generated for this study have been deposited in the Gene Expression Omnibus at NCBI under accession number **GSE213102**. All data analyses were performed in R language using open-source packages. Source codes have been deposited on GitHub and will be provided from R C-D, D A-C, and M P-M upon reasonable request.

# Supplementary Figures and Tables

## Supplementary Figures

**
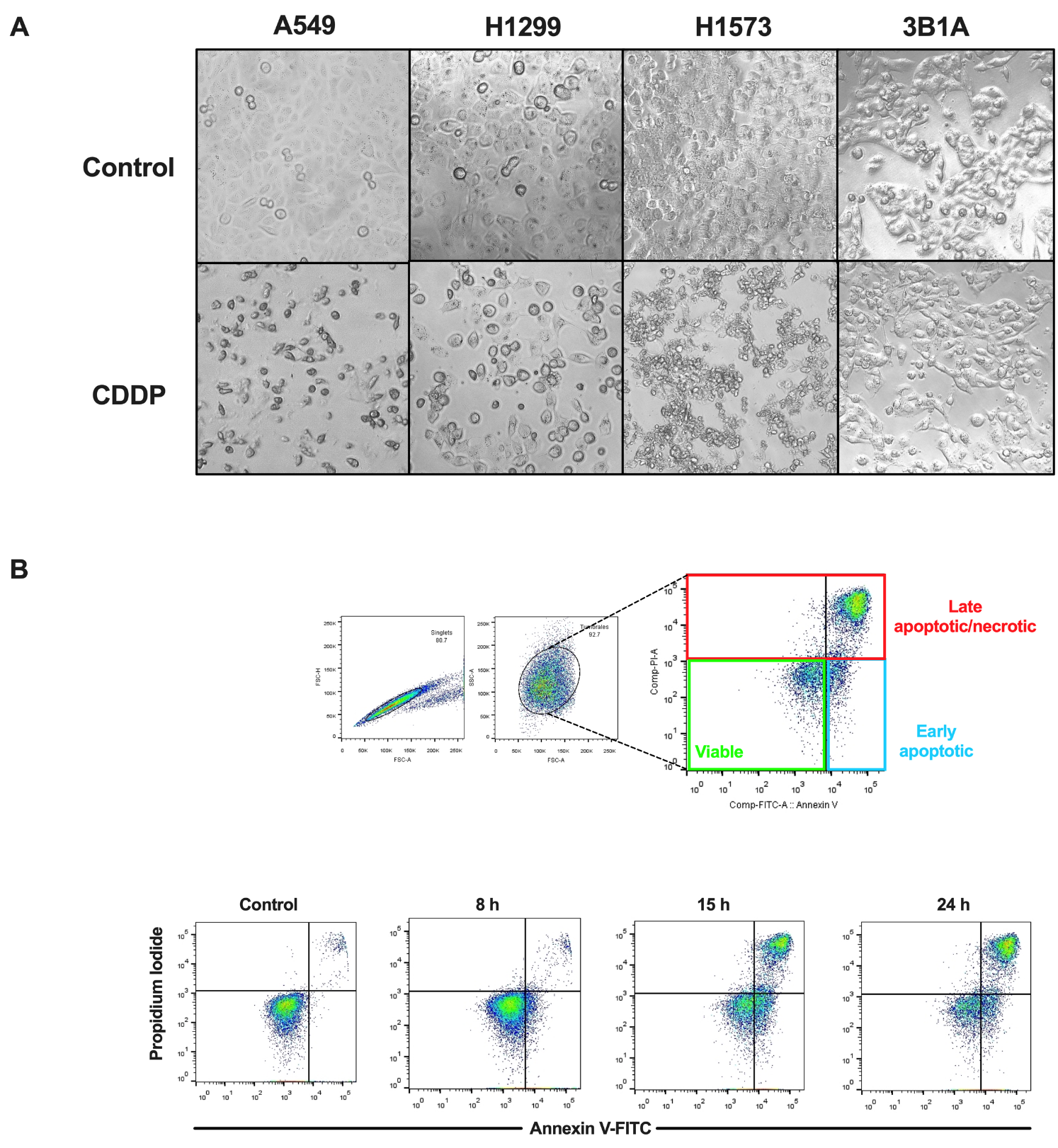
**

**Supplementary Figure 1**. Effect of CDDP in lung adenocarcinoma cell lines. (A) Morphological alterations elicited by CDDP exposure. Representative micrographs comparing control and treated cells are shown. Magnification X200. (B) Representative strategy of analysis in A549 cell line for the quantification of viable, apoptotic, and necrotic cells using flow cytometry along CDDP exposure.


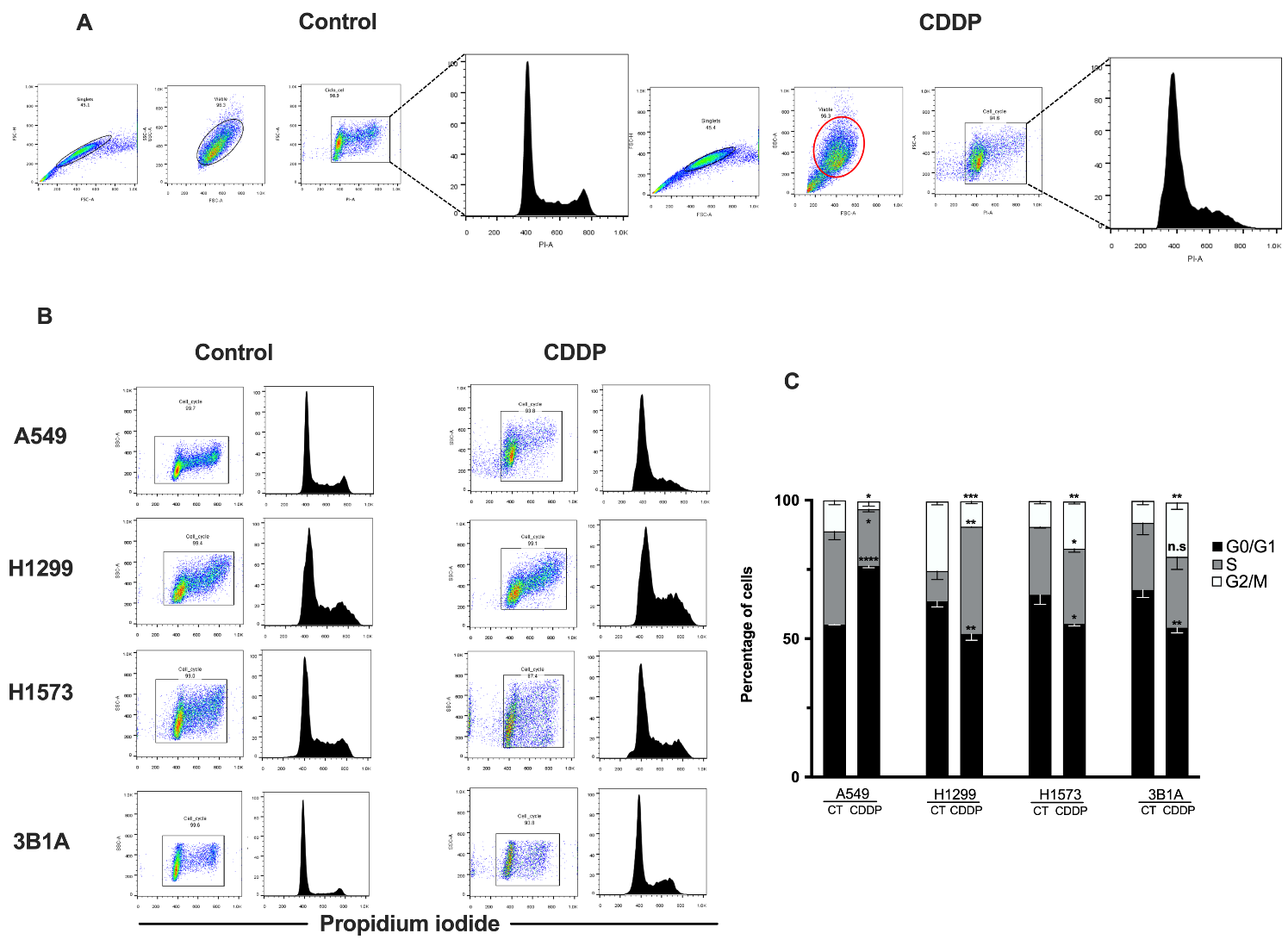


**Supplementary Figure 2**. Distribution of CDDP-DTP cells in the cell cycle phases. (A) Representative strategy of the cell cycle analysis in A549 cell line in control and CDDP-DTP cells is shown. (B) Dot plots and histograms showing the cell cycle phase distribution in control and CDDP-DTP cells from each cell line. (C) Changes in the percentage of cells in the G0/G1, S, and G2/M phases in control (CT) and CDDP-DTP cells (CDDP). Three independent experiments were performed in triplicate. Data are shown as the mean ± SD. The significant difference between control and treated cells is indicated with asterisks (**p* < 0.05, ***p* < 0.01, ****p* < 0.001, *****p* < 0.0001, n.s.= not significant).

**
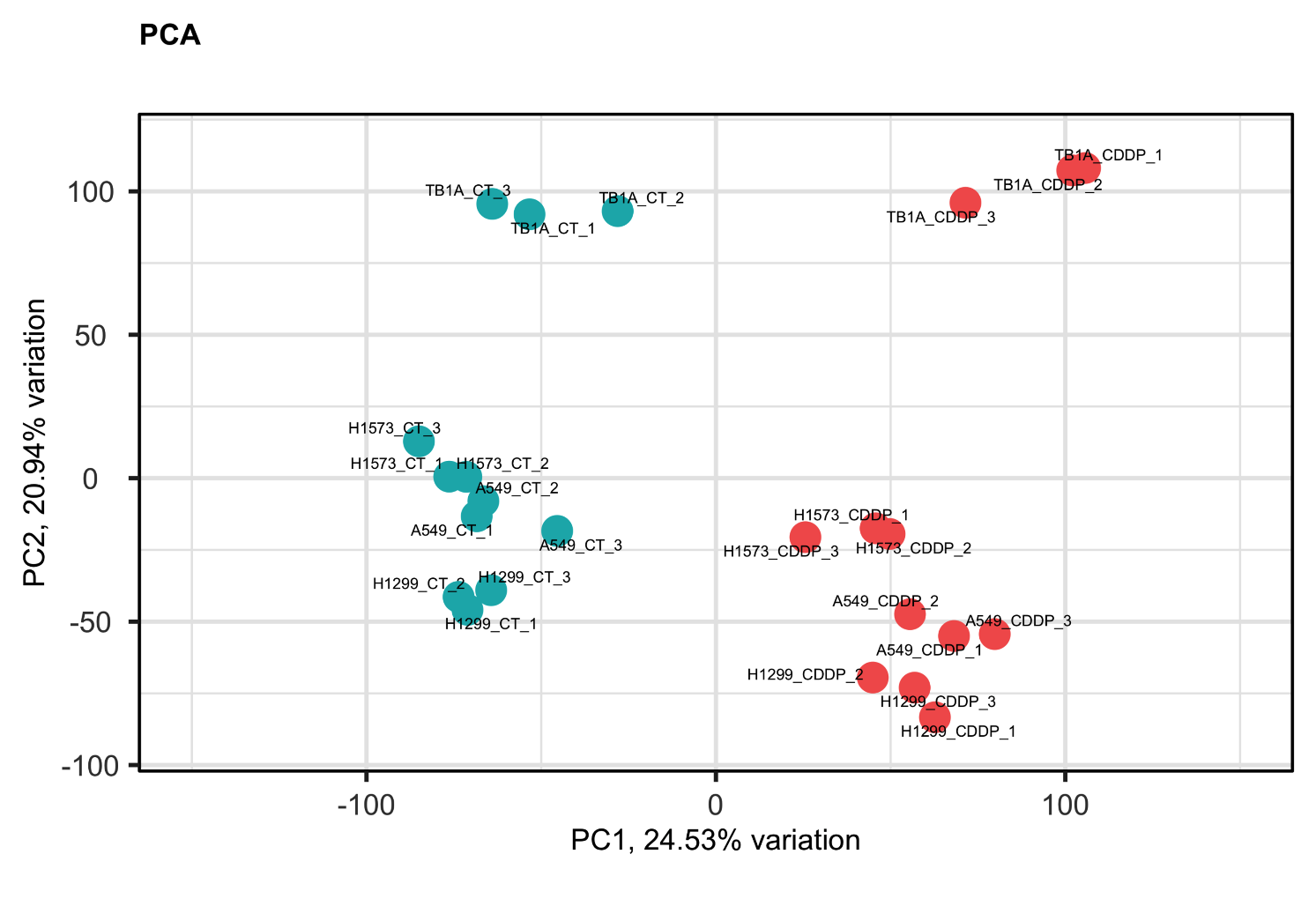
Supplementary Figure 3**. Principal component analysis. Bidimensional plot of the PCA depicting control (green dots) and CDDP-DTP cells (red dots) from RNA-seq datasets.

**
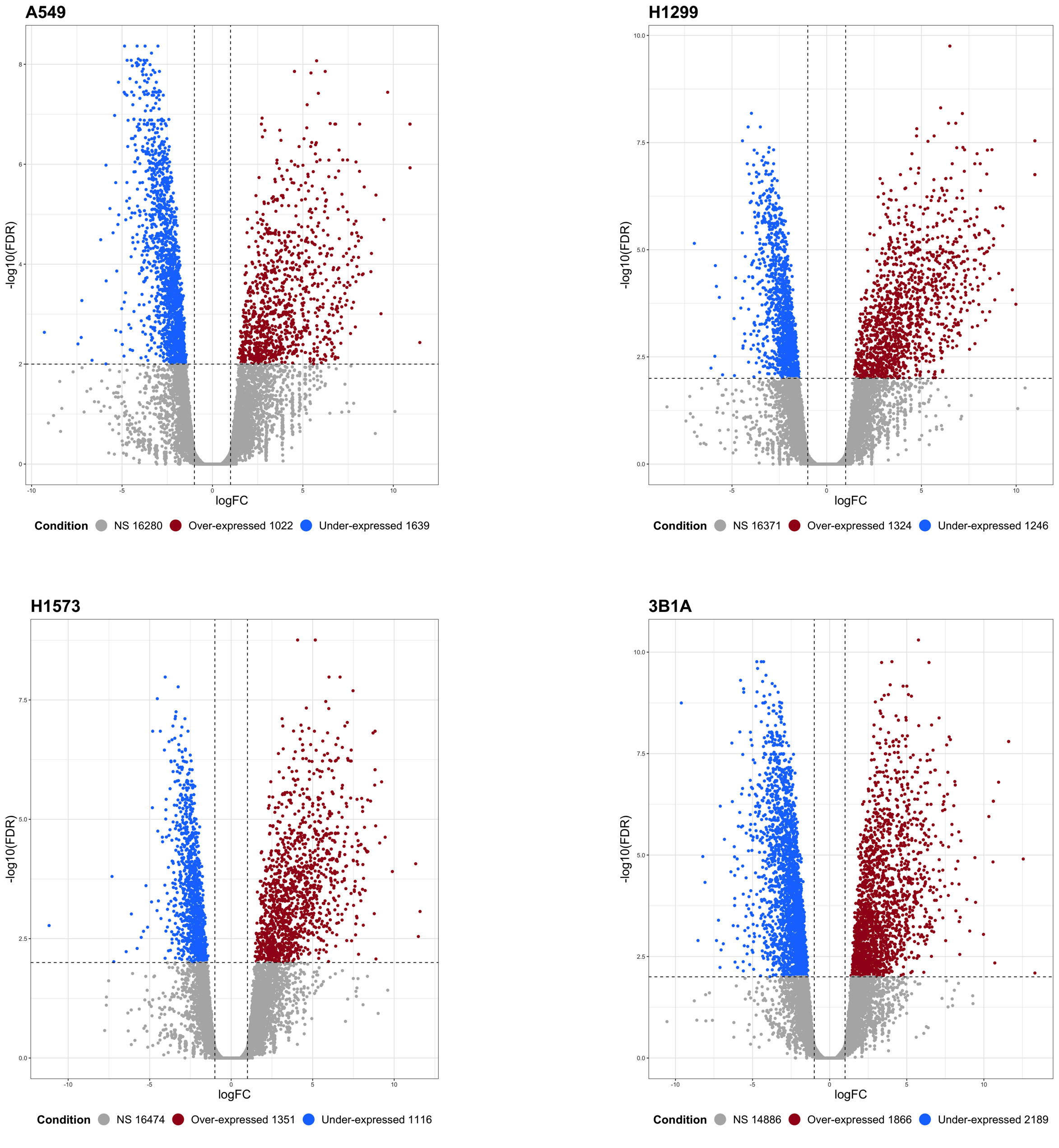
**

**Supplementary Figure 4**. Differentially expressed genes between CDDP-DTP and control cells from each studied cell line. Volcano plots highlight the overexpressed (|log_2_ fold-change| > 1 and *p*-adjusted value < 0.01, red dots), underexpressed (|log_2_ fold-change| < -1 and *p*-adjusted value < 0.01, blue dots) significant, and not significant (NS, gray dots) genes.


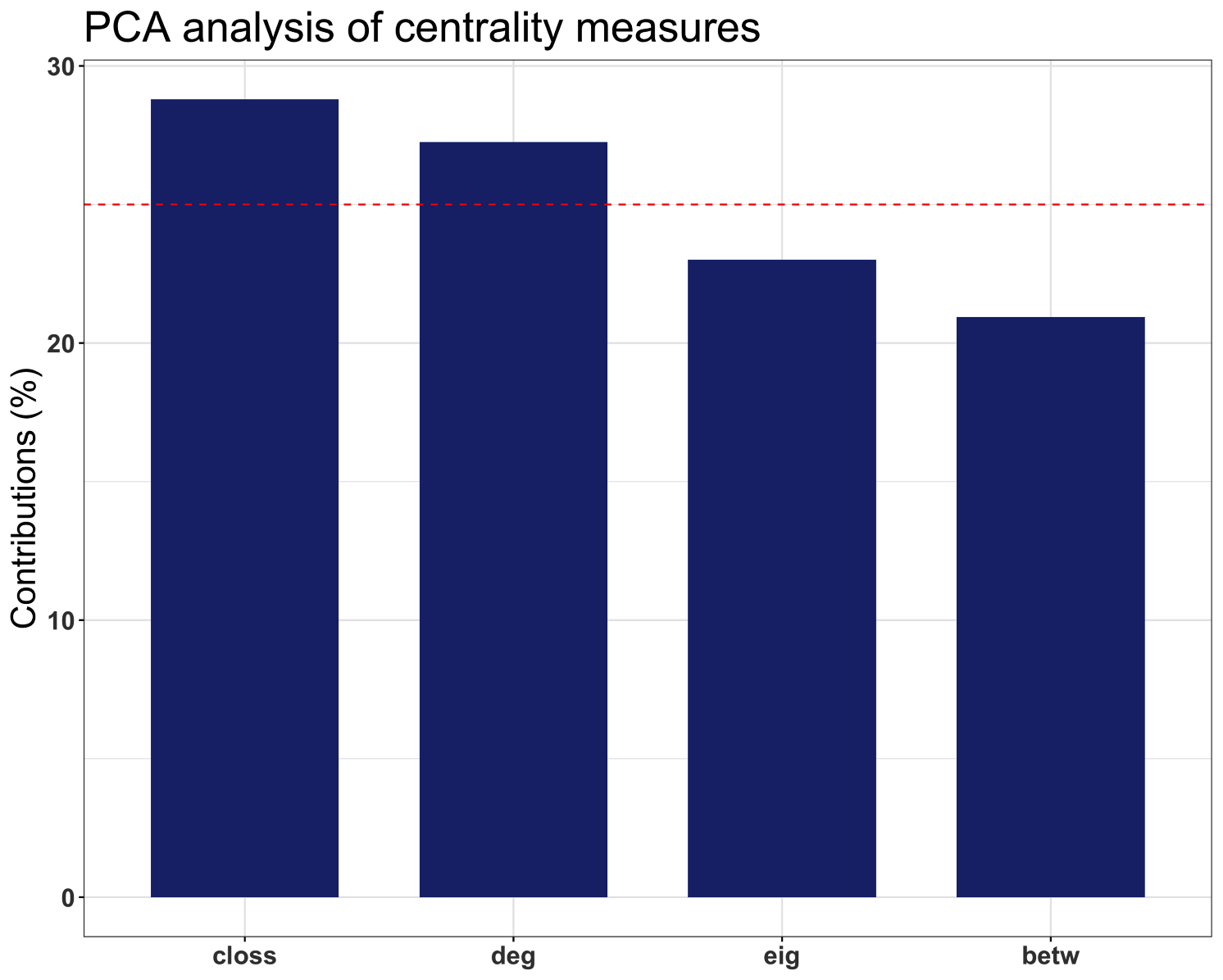


**Supplementary Figure 5**. Contribution of each centrality measurement to the network. Screeplot showing the percentage of variance associated with each centrality variable. Closs = closeness; deg = degree; eig = eigen value; betw = betweeness.


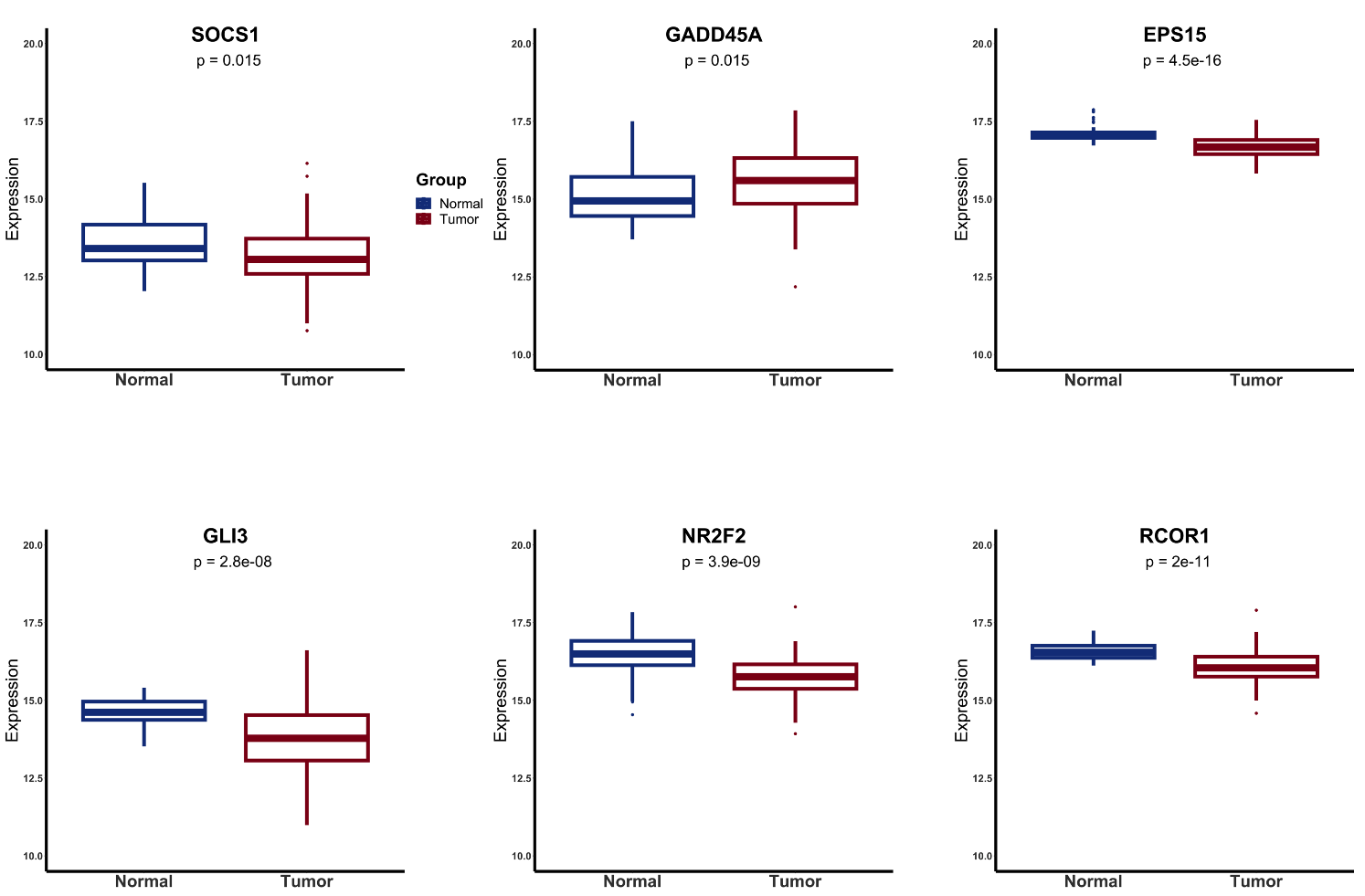


**Supplementary Figure 6**. Relative expression of hub genes associated with CDDP-DTP cells in lung cancer patients. Comparison of the relative expression of each gene between normal adjacent and tumor tissues in the cohort of CDDP-treated patients from the LUAD-TCGA dataset.

## Supplementary Tables

**Supplementary Table 1**. TaqMan probes employed for RT-qPCR assays

| **Gene symbol** | **Gene name** | **TaqMan assay** |
| --- | --- | --- |
| *GADD45A* | Growth Arrest and DNA Damage Inducible Alpha | Hs00169255_m1 |
| *SOCS1* | Suppressor of Cytokine Signaling 1 | Hs00705164_s1 |
| *HEXIM-1* | Hexamethylene Bis-acetamide Inducible 1 | Hs00538918_s1 |
| *HBEGF* | Heparin Binding EGF Like Growth Factor | Hs00181813_m1 |
| *BUB1B* | Mitotic Checkpoint Serine/Threonine Kinase B | Hs01084828_m1 |
| *KIF18A* | Kinesin Family Member 18 | Hs01015428_m1 |
| *ERCC6L* | Excision Repair 6 Like, Spindle Assembly Checkpoint Helicase | Hs_00535177_s1 |
| *NR2F2* | Nuclear Receptor Subfamily 2 Group F Member 2 | Hs04188634_m1 |
| *GAPDH* | Glyceraldehyde-3-Phosphate Dehydrogenase | Hs99999905_m1 |

**Supplementary Table 2**. DsiRNA sequences employed for transfection

| **Name** | **Sequence** | **Sense** |
| --- | --- | --- |
| *hs.Ri.SOCS1.13.1* | *5’ rArGrUrCrArGrUrUrUrArGrGrUrArArUrArArArCrUrUrUAT 3’* | *+* |
|  | *5’ rArUrArArArGrUrUrUrArUrUrArCrCrUrArArArCrUrGrArCrUrUrU 3’* | *-* |
| *hs.Ri.SOCS1.13.2* | *5’ rGrGrUrArArUrArArArCrUrUrUrArUrUrArUrGrArArArGTT 3’* | *+* |
|  | *5’ rArArCrUrUrUrCrArUrArArUrArArArGrUrUrUrArUrUrArCrCrUrA 3’* | *-* |
| *hs.Ri.SOCS1.13.3* | *5’ ArGrUrCrArGrUrUrUrArGrGrUrArArUrArArArCrUrUrArT 3’* | *+* |
|  | *5’ rArUrArArArGrUrUrUrArUrUrArCrCrUrArArArCrUrGrArCrUrUrU 3’* | *-* |
|  |  |  |

**Supplementary Table 3**. Hub nodes from the CDDP-DTPs cells-associated gene network

| **Description** | **Ensembl id** | **Entrez id** | **HUGO symbol** | **logFC** | **FDR** | **Centrality score^a^** | **Community** | **Functional annotation^b^** |
| --- | --- | --- | --- | --- | --- | --- | --- | --- |
| Nuclear Receptor Coactivator 3 | ENSG00000124151 | 8202 | NCOA3 | -2.8187587 | 2.33E-12 | 3.90032867 | 6 | Chromatin remodeling |
| Smad Family Member 3 | ENSG00000166949 | 4088 | SMAD3 | -2.8141401 | 1.69E-11 | 3.90052814 | 6 | Chromatin remodeling |
| Creb Binding Protein | ENSG00000005339 | 1387 | CREBBP | -2.0901902 | 5.03E-09 | 3.90105174 | 6 | Chromatin remodeling |
| Glycogen Synthase Kinase 3 Beta | ENSG00000082701 | 2932 | GSK3B | -2.5221032 | 1.06E-10 | 3.90185897 | 16 | Canonical beta-catenin pathway |
| Sin3 Transcription Regulator Family Member A | ENSG00000169375 | 25942 | SIN3A | -3.0255246 | 6.72E-13 | 3.90213947 | 6 | Chromatin remodeling |
| Histone Deacetylase 4 | ENSG00000068024 | 9759 | HDAC4 | -2.6477618 | 2.62E-11 | 3.90272916 | 6 | Chromatin remodeling |
| Galectin 4 | ENSG00000171747 | 3960 | LGALS4 | 3.04402633 | 7.09E-08 | 3.90374766 | 6 | Chromatin remodeling |
| Ccctc-Binding Factor | ENSG00000102974 | 10664 | CTCF | -2.3913772 | 6.08E-12 | 3.90381097 | 6 | Chromatin remodeling |
| Actin Alpha 1, Skeletal Muscle | ENSG00000143632 | 58 | ACTA1 | 4.47486262 | 4.77E-09 | 3.90413061 | 2 | Other |
| Insulin Like Growth Factor 1 Receptor | ENSG00000140443 | 3480 | IGF1R | -2.3128209 | 4.35E-11 | 3.90451409 | 3 | GTPase and lipid kinases metabolism |
| Chromodomain Helicase Dna Binding Protein 7 | ENSG00000171316 | 55636 | CHD7 | -2.3243873 | 1.25E-09 | 3.90472236 | 6 | Chromatin remodeling |
| Transcription Factor 7 Like 2 | ENSG00000148737 | 6934 | TCF7L2 | -3.6279697 | 2.24E-12 | 3.90492577 | 6 | Chromatin remodeling |
| Tbl1x Receptor 1 | ENSG00000177565 | 79718 | TBL1XR1 | -2.592769 | 5.51E-10 | 3.90509246 | 6 | Chromatin remodeling |
| Lysine Demethylase 6a | ENSG00000147050 | 7403 | KDM6A | -2.4777775 | 9.49E-10 | 3.90531514 | 6 | Chromatin remodeling |
| MYST Histone Acetyltransferase (Monocytic Leukemia) 4 | ENSG00000156650 | 23522 | MYST4 | -3.118998 | 1.465033E-12 | 3.9055525 | Other | Other |
| Mllt3 Super Elongation Complex Subunit | ENSG00000171843 | 4300 | MLLT3 | -3.0459279 | 8.66E-12 | 3.90571311 | 6 | Chromatin remodeling |
| Beta-Transducin Repeat Containing E3 Ubiquitin Protein Ligase | ENSG00000166167 | 8945 | BTRC | -2.6075646 | 5.96E-11 | 3.9058289 | 16 | Canonical beta-catenin pathway |
| Set Domain Containing 2, Histone Lysine Methyltransferase | ENSG00000181555 | 29072 | SETD2 | -2.1073625 | 1.29E-08 | 3.90618339 | 6 | Chromatin remodeling |
| At-Rich Interaction Domain 4b | ENSG00000054267 | 51742 | ARID4B | -2.7080477 | 5.52E-11 | 3.90639129 | 6 | Chromatin remodeling |
| Nuclear Receptor Subfamily 3 Group C Member 1 | ENSG00000113580 | 2908 | NR3C1 | -2.6661124 | 5.03E-10 | 3.90656366 | 6 | Chromatin remodeling |
| Recombination Signal Binding Protein For Immunoglobulin Kappa J Region | ENSG00000168214 | 3516 | RBPJ | -2.1712643 | 9.26E-10 | 3.90665413 | 6 | Chromatin remodeling |
| Nuclear Factor Kappa B Subunit 1 | ENSG00000109320 | 4790 | NFKB1 | -2.0427344 | 2.98E-09 | 3.90681121 | 16 | Canonical beta-catenin pathway |
| Transducin Beta Like 1 X-Linked | ENSG00000101849 | 6907 | TBL1X | -2.9873526 | 5.88E-13 | 3.90699536 | 6 | Chromatin remodeling |
| Activating Transcription Factor 3 | ENSG00000162772 | 467 | ATF3 | 3.71331663 | 5.00E-12 | 3.90727729 | 16 | Canonical beta-catenin pathway |
| Transcription Factor 12 | ENSG00000140262 | 6938 | TCF12 | -2.0768911 | 2.74E-09 | 3.90767089 | 6 | Chromatin remodeling |
| C-Terminal Binding Protein 2 | ENSG00000175029 | 1488 | CTBP2 | -2.2470137 | 3.44E-11 | 3.90772633 | 6 | Chromatin remodeling |
| Gli Family Zinc Finger 3 | ENSG00000106571 | 2737 | GLI3 | -3.7617425 | 3.04E-11 | 3.90811984 | 6 | Chromatin remodeling |
| Ets Variant Transcription Factor 6 | ENSG00000139083 | 2120 | ETV6 | -2.3679016 | 2.75E-09 | 3.90824067 | 6 | Chromatin remodeling |
| Phd Finger Protein 20 | ENSG00000025293 | 51230 | PHF20 | -2.0560567 | 6.88E-10 | 3.90831802 | 6 | Chromatin remodeling |
| Fyn Proto-Oncogene, Src Family Tyrosine Kinase | ENSG00000010810 | 2534 | FYN | -3.5042792 | 7.40E-10 | 3.90839011 | 3 | GTPase and lipid kinases metabolism |
| Mitogen-Activated Protein Kinase Kinase Kinase 5 | ENSG00000197442 | 4217 | MAP3K5 | -2.8729773 | 4.24E-09 | 3.90841113 | 8 | Vesicle transport and apoptosis |
| Protein Phosphatase 2 Regulatory Subunit B'epsilon | ENSG00000154001 | 5529 | PPP2R5E | -2.6190568 | 1.94E-11 | 3.90859492 | 3 | GTPase and lipid kinases metabolism |
| Mediator Complex Subunit 13l | ENSG00000123066 | 23389 | MED13L | -2.8933219 | 1.10E-10 | 3.90871047 | 6 | Chromatin remodeling |
| Rest Corepressor 1 | ENSG00000089902 | 23186 | RCOR1 | -2.3151021 | 9.50E-12 | 3.90887646 | 6 | Chromatin remodeling |
| Vav Guanine Nucleotide Exchange Factor 2 | ENSG00000160293 | 7410 | VAV2 | -2.2244349 | 2.95E-11 | 3.90896462 | 3 | GTPase and lipid kinases metabolism |
| Rho Guanine Nucleotide Exchange Factor 7 | ENSG00000102606 | 8874 | ARHGEF7 | -2.7587498 | 2.83E-12 | 3.9090623 | 3 | GTPase and lipid kinases metabolism |
| At-Rich Interaction Domain 1b | ENSG00000049618 | 57492 | ARID1B | -2.6687638 | 1.31E-11 | 3.90923886 | 6 | Chromatin remodeling |
| Yeats Domain Containing 2 | ENSG00000163872 | 55689 | YEATS2 | -1.8618577 | 7.17E-08 | 3.90976788 | 6 | Chromatin remodeling |
| Retinoid X Receptor Alpha | ENSG00000186350 | 6256 | RXRA | -2.5498021 | 1.18E-10 | 3.90982102 | 6 | Chromatin remodeling |
| Nuclear Receptor Binding Set Domain Protein 1 | ENSG00000165671 | 64324 | NSD1 | -2.0625503 | 3.95E-09 | 3.90990461 | 6 | Chromatin remodeling |
| Zinc Finger Mynd-Type Containing 8 | ENSG00000101040 | 23613 | ZMYND8 | -3.8161284 | 6.72E-13 | 3.91018925 | 12 | Cell cycle control |
| Euchromatic Histone Lysine Methyltransferase 1 | ENSG00000181090 | 79813 | EHMT1 | -2.1402626 | 2.78E-11 | 3.91028751 | 6 | Chromatin remodeling |
| Rho Associated Coiled-Coil Containing Protein Kinase 2 | ENSG00000134318 | 9475 | ROCK2 | -2.5355894 | 1.56E-09 | 3.91032328 | 3 | GTPase and lipid kinases metabolism |
| Cullin 2 | ENSG00000108094 | 8453 | CUL2 | -1.8038915 | 2.89E-08 | 3.91063006 | 23 | Other |
| Tet Methylcytosine Dioxygenase 1 | ENSG00000138336 | 80312 | TET1 | -3.3788635 | 1.04E-08 | 3.91069451 | 6 | Chromatin remodeling |
| Nuclear Receptor Subfamily 2 Group F Member 2 | ENSG00000185551 | 7026 | NR2F2 | -2.5207117 | 3.52E-10 | 3.91069557 | 6 | Chromatin remodeling |
| Glucocorticoid Receptor DNA-Binding Factor 1 | ENSG00000160007 | 2909 | GRLF1 | -2.781841 | 2.258417E-12 | 3.91082443 | Other | Other |
| Golgi Phosphoprotein 3 | ENSG00000113384 | 64083 | GOLPH3 | -2.5376517 | 2.26E-12 | 3.91095368 | 8 | Vesicle transport and apoptosis |
| At-Rich Interaction Domain 2 | ENSG00000189079 | 196528 | ARID2 | -2.8217547 | 1.18E-10 | 3.91100209 | 6 | Chromatin remodeling |
| Mastermind Like Transcriptional Coactivator 2 | ENSG00000184384 | 84441 | MAML2 | -3.3896987 | 1.03E-09 | 3.91111817 | 6 | Chromatin remodeling |
| Frizzled Class Receptor 2 | ENSG00000180340 | 2535 | FZD2 | -2.6139618 | 1.04E-09 | 3.91123359 | 16 | Canonical beta-catenin pathway |
| Tiam Rac1 Associated Gef 1 | ENSG00000156299 | 7074 | TIAM1 | -2.757247 | 2.68E-10 | 3.91127323 | 3 | GTPase and lipid kinases metabolism |
| Suppressor Of Cytokine Signaling 1 | ENSG00000185338 | 8651 | SOCS1 | 3.32256452 | 6.64E-09 | 3.91127547 | 3 | GTPase and lipid kinases metabolism |
| Mllt10 Histone Lysine Methyltransferase Dot1l Cofactor | ENSG00000078403 | 8028 | MLLT10 | -2.481167 | 1.09E-12 | 3.91135249 | 6 | Chromatin remodeling |
| Epidermal Growth Factor Receptor Pathway Substrate 15 | ENSG00000085832 | 2060 | EPS15 | -2.4996284 | 2.83E-10 | 3.9114377 | 8 | Vesicle transport and apoptosis |
| Nuclear Receptor Coactivator 2 | ENSG00000140396 | 10499 | NCOA2 | -3.9572096 | 5.27E-11 | 3.91170197 | 6 | Chromatin remodeling |
| Dual Specificity Tyrosine Phosphorylation Regulated Kinase 1a | ENSG00000157540 | 1859 | DYRK1A | -2.1144252 | 1.08E-08 | 3.91178127 | 6 | Chromatin remodeling |
| Zinc Finger Protein 148 | ENSG00000163848 | 7707 | ZNF148 | -2.5935054 | 1.43E-09 | 3.91221887 | 6 | Chromatin remodeling |
| Itchy E3 Ubiquitin Protein Ligase | ENSG00000078747 | 83737 | ITCH | -2.0184718 | 1.23E-07 | 3.9123122 | 9 | Protein ubiquitylation |
| Nck Adaptor Protein 1 | ENSG00000158092 | 4690 | NCK1 | -2.9984795 | 1.75E-12 | 3.91256649 | 3 | GTPase and lipid kinases metabolism |
| Tripartite Motif Containing 24 | ENSG00000122779 | 8805 | TRIM24 | -2.1740478 | 4.28E-09 | 3.9126863 | 6 | Chromatin remodeling |
| Ino80 Complex Atpase Subunit | ENSG00000128908 | 54617 | INO80 | -2.3545065 | 6.49E-12 | 3.91271529 | 6 | Chromatin remodeling |
| Set Domain Containing 5 | ENSG00000168137 | 55209 | SETD5 | -2.2028825 | 1.10E-10 | 3.91272984 | 6 | Chromatin remodeling |
| Ph Domain And Leucine Rich Repeat Protein Phosphatase 1 | ENSG00000081913 | 23239 | PHLPP1 | -3.786795 | 1.36E-11 | 3.91276148 | 2 | Other |
| Nuclear Receptor Coactivator 6 | ENSG00000198646 | 23054 | NCOA6 | -2.1083674 | 1.21E-08 | 3.91278123 | 6 | Chromatin remodeling |
| G Protein Subunit Alpha 12 | ENSG00000146535 | 2768 | GNA12 | -2.4207562 | 1.51E-11 | 3.91292964 | 3 | GTPase and lipid kinases metabolism |
| Activity Dependent Neuroprotector Homeobox | ENSG00000101126 | 23394 | ADNP | -2.1449552 | 4.02E-11 | 3.91296997 | 6 | Chromatin remodeling |
| Hivep Zinc Finger 1 | ENSG00000095951 | 3096 | HIVEP1 | -2.3907083 | 1.11E-08 | 3.91299374 | 19 | Other |
| Cullin 1 | ENSG00000055130 | 8454 | CUL1 | -2.3721997 | 4.05E-12 | 3.91323522 | 6 | Chromatin remodeling |
| Ldl Receptor Related Protein 6 | ENSG00000070018 | 4040 | LRP6 | -2.6271881 | 9.04E-11 | 3.91323777 | 16 | Canonical beta-catenin pathway |
| Nuclear Receptor Coactivator 1 | ENSG00000084676 | 8648 | NCOA1 | -2.7261226 | 3.85E-10 | 3.91325633 | 6 | Chromatin remodeling |
| Growth Arrest And Dna Damage Inducible Alpha | ENSG00000116717 | 1647 | GADD45A | 3.05104643 | 3.59E-13 | 3.91340394 | 16 | Canonical beta-catenin pathway |
| F-Box And Wd Repeat Domain Containing 11 | ENSG00000072803 | 23291 | FBXW11 | -2.7706487 | 2.31E-10 | 3.91340606 | 16 | Canonical beta-catenin pathway |
| Patched 1 | ENSG00000185920 | 5727 | PTCH1 | -3.6789584 | 4.88E-09 | 3.91345186 | 16 | Canonical beta-catenin pathway |
| Jumonji And At-Rich Interaction Domain Containing 2 | ENSG00000008083 | 3720 | JARID2 | -4.3335919 | 3.59E-13 | 3.91371692 | 6 | Chromatin remodeling |
| Tet Methylcytosine Dioxygenase 3 | ENSG00000187605 | 200424 | TET3 | -2.2953557 | 2.26E-10 | 3.91386575 | 6 | Chromatin remodeling |
| Myosin Ixb | ENSG00000099331 | 4650 | MYO9B | -2.039598 | 3.50E-10 | 3.91405286 | 3 | GTPase and lipid kinases metabolism |
| Bromodomain Adjacent To Zinc Finger Domain 2b | ENSG00000123636 | 29994 | BAZ2B | -3.1935704 | 1.55E-10 | 3.91413966 | 6 | Chromatin remodeling |
| Grb10 Interacting Gyf Protein 2 | ENSG00000204120 | 26058 | GIGYF2 | -2.2757488 | 5.74E-11 | 3.91451293 | 12 | Cell cycle control |
| Dishevelled Associated Activator Of Morphogenesis 1 | ENSG00000100592 | 23002 | DAAM1 | -2.7748484 | 5.85E-07 | 3.91454402 | 8 | Vesicle transport and apoptosis |
| Phosphatidylinositol-4,5-Bisphosphate 3-Kinase Catalytic Subunit Beta | ENSG00000051382 | 5291 | PIK3CB | -3.017382 | 5.88E-13 | 3.91464045 | 3 | GTPase and lipid kinases metabolism |
| Solute Carrier Family 2 Member 1 | ENSG00000117394 | 6513 | SLC2A1 | -2.5688982 | 1.37E-08 | 3.91503135 | 23 | Other |
| Transcription Factor 20 | ENSG00000100207 | 6942 | TCF20 | -2.2107768 | 9.98E-11 | 3.91507919 | 6 | Chromatin remodeling |
| Transforming Growth Factor Beta Receptor 3 | ENSG00000069702 | 7049 | TGFBR3 | -4.1584234 | 1.05E-09 | 3.91514446 | 7 | Other |
| Chromodomain Y Like | ENSG00000153046 | 9425 | CDYL | -3.1097543 | 1.08E-12 | 3.91532099 | 6 | Chromatin remodeling |
| Myelin Basic Protein | ENSG00000197971 | 4155 | MBP | -2.8004101 | 4.60E-11 | 3.91532205 | 14 | Other |
| Tata-Box Binding Protein Associated Factor 4 | ENSG00000130699 | 6874 | TAF4 | -2.426617 | 9.96E-11 | 3.91578109 | 6 | Chromatin remodeling |
| Gata Zinc Finger Domain Containing 2b | ENSG00000143614 | 57459 | GATAD2B | -2.7079263 | 1.86E-10 | 3.91584656 | 6 | Chromatin remodeling |
| Transforming Growth Factor Beta Receptor 2 | ENSG00000163513 | 7048 | TGFBR2 | -3.4042692 | 5.87E-09 | 3.91589907 | 9 | Protein ubiquitylation |
| Ras Homolog Family Member F, Filopodia Associated | ENSG00000139725 | 54509 | RHOF | -2.2565458 | 9.51E-12 | 3.91603893 | 3 | GTPase and lipid kinases metabolism |
| Swi/Snf-Related, Matrix-Associated Actin-Dependent Regulator Of Chromatin, Subfamily A, Containing Dead/H Box 1 | ENSG00000163104 | 56916 | SMARCAD1 | -2.428476 | 1.34E-07 | 3.9163933 | 6 | Chromatin remodeling |
| Nuclear Factor I C | ENSG00000141905 | 4782 | NFIC | -2.322691 | 5.48E-11 | 3.91671119 | 22 | Other |
| Heparin Binding Egf Like Growth Factor | ENSG00000113070 | 1839 | HBEGF | 3.86394999 | 1.98E-11 | 3.91671424 | 3 | GTPase and lipid kinases metabolism |
| Ecotropic Viral Integration Site 5 | ENSG00000067208 | 7813 | EVI5 | -2.486357 | 9.26E-10 | 3.91688782 | 16 | Canonical beta-catenin pathway |
| Hexim P-Tefb Complex Subunit 1 | ENSG00000186834 | 10614 | HEXIM1 | 2.53014104 | 5.20E-10 | 3.91692021 | 6 | Chromatin remodeling |
| Ras P21 Protein Activator 2 | ENSG00000155903 | 5922 | RASA2 | -2.8972431 | 1.18E-10 | 3.91735466 | 3 | GTPase and lipid kinases metabolism |
| Activating Transcription Factor 7 Interacting Protein | ENSG00000171681 | 55729 | ATF7IP | -2.4725922 | 1.62E-09 | 3.91747505 | 6 | Chromatin remodeling |
| Phd Finger Protein 2 | ENSG00000197724 | 5253 | PHF2 | -2.6393443 | 1.77E-12 | 3.91752158 | 6 | Chromatin remodeling |
| Protein Kinase C Epsilon | ENSG00000171132 | 5581 | PRKCE | -2.583474 | 7.59E-09 | 3.9176373 | 5 | Other |

^a^ Centrality score value calculated as the -log10 closeness value.

^b^ Main enriched GO term associated with the functional annotation of the community
